# Comparative Analysis of Tenogenic Gene Expression in Tenocyte-Derived Induced Pluripotent Stem Cells and Bone Marrow-Derived Mesenchymal Stem Cells in Response to Biochemical and Biomechanical Stimuli

**DOI:** 10.1155/2021/8835576

**Published:** 2021-01-13

**Authors:** Feikun Yang, Dean W. Richardson

**Affiliations:** Department of Clinical Studies New Bolton Center, School of Veterinary Medicine, University of Pennsylvania, 382 West Street Road, Kennett Square PA 19348, USA

## Abstract

The tendon is highly prone to injury, overuse, or age-related degeneration in both humans and horses. Natural healing of injured tendon is poor, and cell-based therapeutic treatment is still a significant clinical challenge. In this study, we extensively investigated the expression of tenogenic genes in equine bone marrow mesenchymal stem cells (BMSCs) and tenocyte-derived induced pluripotent stem cells (teno-iPSCs) stimulated by growth factors (TGF-*β*3 and BMP12) combined with ectopic expression of tenogenic transcription factor MKX or cyclic uniaxial mechanical stretch. Western blotting revealed that TGF-*β*3 and BMP12 increased the expression of transcription factors SCX and MKX in both cells, but the tenocyte marker tenomodulin (TNMD) was detected only in BMSCs and upregulated by either inducer. On the other hand, quantitative real-time PCR showed that TGF-*β*3 increased the expression of *EGR1*, *COL1A2*, *FMOD*, and *TNC* in BMSCs and *SCX*, *COL1A2*, *DCN*, *FMOD*, and *TNC* in teno-iPSCs. BMP12 treatment elevated *SCX*, *MKX*, *DCN*, *FMOD*, and *TNC* in teno-iPSCs. Overexpression of MKX increased *SCX*, *DCN*, *FMOD*, and *TNC* in BMSCs and *EGR1*, *COL1A2*, *DCN*, *FMOD*, and *TNC* in teno-iPSCs; TGF-*β*3 further enhanced *TNC* in BMSCs. Moreover, mechanical stretch increased *SCX*, *EGR1*, *DCN*, *ELN*, and *TNC* in BMSCs and *SCX*, *MKX*, *EGR1*, *COL1A2*, *DCN*, *FMOD*, and *TNC* in teno-iPSCs; TGF-*β*3 tended to further elevate *SCX*, *ELN*, and *TNC* in BMSCs and *SCX*, *MKX*, *COL1A2*, *DCN*, and *TNC* in teno-iPSCs, while BMP12 further uptrended the expression of *SCX* and *DCN* in BMSCs and *DCN* in teno-iPSCs. Additionally, the aforementioned tenogenic inducers also affected the expression of signaling regulators *SMAD7*, *ETV4*, and *SIRT1* in BMSCs and teno-iPSCs. Taken together, our data demonstrate that, in respect to the tenocyte-lineage-specific gene expression, BMSCs and teno-iPSCs respond differently to the tenogenic stimuli, which may affect the outcome of their application in tendon repair or regeneration.

## 1. Introduction

The tendon is a hypovascular tissue transmitting force from the muscle to the bone. It is subject to high risk of injury from acute trauma, overuse, or age-related degeneration. The limited natural healing capacity and the poor functional outcomes of tendon repair are pushing the search for more effective regenerative approaches [1]. Stem cells, including embryonic stem cells (ESCs), induced pluripotent stem cells (iPSCs), and mesenchymal stem cells (MSCs), possess tenogenic differentiation capacity and have been proposed for tendon repair and regeneration [2]. For example, tendon stem/progenitor cells (TSPCs) showed high capacity to form a tendon-like tissue *in vitro* and *in vivo* [3, 4] and were suggested to be a better cell source for the treatment of tendon disorders than other types of stem cells [5]. However, their application is limited due to the relatively low number within the whole tendon cell population and the loss of phenotype following *in vitro* expansion [6]. Bone marrow-derived MSCs (BMSCs) have been extensively studied for tendon repair in humans and horses, but the direct use of undifferentiated BMSCs for clinical practice is still debatable partly because of the formation of ectopic bone- or cartilage-like structure at the target sites [7]. iPSCs showed great promise as an emerging cell source for tendon repair [8–10], however, the potential of oncogenic formation is always a concern and more extensive studies are needed before their clinical translation [11]. One alternative way to improve the stem cell-based tendon therapies will be, prior to stem cell implantation, predirecting stem cells toward the tenogenic lineage *in vitro* by using biological (including transcription factors, growth factors, and microenvironment) and biomechanical cues.

Transforming growth factor beta (TGF-*β*) superfamily of cytokines, including TGF-*β* subfamily (TGF-*β*1, TGF-*β*2, and TGF-*β*3), bone morphogenic proteins/growth differentiation factors (BMP/GDFs), and activin/inhibin, plays crucial roles in tendon development, homeostasis, and pathogenesis [12–15]. In TGF-*β*2- and/or TGF-*β*3-deficient mouse embryos, loss of tendons and ligaments was observed throughout the body, along with no detectable signals of tenocyte-related genes *SCX*, *TNMD*, and *COL1A1* [13]. Targeted deletion of the TGF-*β* type 2 receptor (Tgf*β*r2) in tenocytes did not disrupt the tendon differentiation function and growth during embryonic development but destroyed the differentiation markers *SCX*, *TNMD*, and *COL1A1* shortly after birth and reverted the mutant cells to a more progenitor-like state [15]. Moreover, *in vitro* studies revealed that TGF-*β*2 was able to induce *SCX* expression in embryonic fibroblast cells, mesenchymal stem cell line C3H10T1/2, and mouse limb bud in an organ culture [13]. TGF-*β*3 was reported to promote tenogenic gene expression in different types of stem cells [14, 16, 17] but its use in iPSCs is very limited. Only one laboratory reported that, with TGF-*β*3 stimulation, equine iPSCs had a reduced tendon differentiation capacity compared to ESCs [18].

Although BMP/GDFs were originally named for their ability to induce bone formation, the family members BMP12 (GDF7), BMP13 (GDF6), and BMP14 (GDF5) were shown to play important roles in tendon/ligament maintenance and repair [12]. Compared to those in wildtype littermates, the tendons in BMP14^−/−^ and BMP13^−/−^ mice showed similar defects on collagen production and mechanical properties [19, 20]. In BMP12-deficient mice, while the expression of fibrillar collagens and tendon proteoglycans was not affected, the Achilles tendon exhibited a shift towards smaller diameter fibrils that resulted in a small but significant reduction in mean fibril diameter [21]. In *in vitro* studies, although BMP12 has been shown to induce the expression of *TNMD* and *DCN* in equine BMSCs [22] and amniotic fluid-derived MSCs [23], the expression of *SCX* and *TNMD* in canine adipose-derived stromal cells (ASCs) [24], and the expression of *SCX* and *MKX* in human ASCs [25], its application in iPSCs has not been reported yet.

Biophysical force and at least three transcription factors (SCX, MKX, and *EGR1*) are known to be essential for normal tendon development. Previous works from our laboratory and others have demonstrated that mechanical loading and/or ectopic expression of those transcription factors are able to induce the expression of some tenocyte-related genes in MSCs and iPSCs [26–29]. However, it is still largely unknown how a cell behaves under the circumstances of tenogenic stimulation, and the biomarkers specific for tenocyte lineage are also very limited. It is therefore necessary to examine the activities of a great number of tendon-related genes in tenogenic differentiating cells. In the present study, we aimed to compare the *in vitro* tenogenic differentiation capacity of equine tenocyte-derived iPSCs (teno-iPSCs) and BMSCs induced by bioactive molecules TGF-*β*3 and BMP12 combined with ectopic expression of Mohawk or cyclic uniaxial mechanical stretch. The expression of tenogenic transcription factors (*SCX*, *MKX*, and *EGR1*), tendon extracellular matrix genes (*COL1A2*, decorin (*DCN*), elastin (*ELN*), fibromodulin (*FMOD*), and tenascin C (*TNC*)), and signaling regulators (*SMAD7*, *ETV4*, and Sirtuin1 (*SIRT1*)) was determined. Our goal was to provide valuable information for ongoing and future stem cell-based regenerative treatments of tendon injuries.

## 2. Materials and Methods

### 2.1. Cell Culture

Isolation and culture of equine BMSCs were described in our previous study [28]. Briefly, bone marrow aspirates were washed twice with PBS followed by two more washes with basic medium (DMEM/F12 (Invitrogen) with 10% FCS (Gemini) and 1x antibiotics (Gibco)), and then resuspended and cultured in BMSC growth medium (basic medium plus 4 ng/mL bFGF) at 37°C, 5% CO_2_. After 72 hours, cells were thoroughly washed with PBS, and fresh medium was added with a change of every 2-3 days. Upon reaching 80-90% confluency, cells (P0) were dissociated with 0.25% trypsin-EDTA and further expanded at a density of 1-2 × 10^5^ cells/cm^2^. BMSCs at passages 2-5 were used for experiments. Characterization of mesenchymal stem cell was carried out by flow cytometry with positive expression of CD29, CD44, CD90, CD105, and MHC-I and with negative expression of CD45, CD79, and MHC-II. The multipotency of BMSCs was confirmed by *in vitro* trilineage differentiation using protocols described in our previous work [28] (Supplemental Figure 1).

Generation and multilineage differentiation of teno-iPSCs were also reported in our previous work [28]. Briefly, tenocytes were infected with pHAGE-STEMCCA lentiviruses expressing mouse Oct3/4, SOX2, Klf4, and c-Myc in basic medium for 30 h, and then transferred to mitomycin C inactivated MEF feeder cells in iPSC medium (DMEM containing 10% FCS, 1× NEAA, 1× L-glutamine, 1× sodium pyruvate, 0.055 mM beta-mercaptoethanol, 1000 U/mL of LIF, and 1× antibiotic/antimycotic solution). Medium was replaced every other day. About 10–15 days, individual colonies were manually picked, trypsinized, and further expanded. At passages 3–5, cells were switched to and maintained in feeder-free StemFlex™ medium (Fisher Scientific) and characterized for multilineage differentiation capacity.

### 2.2. Lentiviral Infection

GFP and equine Mohawk gene were subcloned into replication-defective lentiviral vector pHAGE in which these two genes were separated by IRES (internal ribosome entry site) element. Lentiviruses expressing GFP (lenti-GFP) alone or MKX and GFP (lenti-MKX) were produced in 293T packaging cells, and supernatant containing the viral particles passed through a Millex-HV 0.45 *μ*m PVDF filter (Millipore, Ireland). Cells seeded on 35 mm culture plates at a density of 20,000 cells/cm^2^ the day before infection were exposed to 1 : 1 dilution of filtered viral supernatant in the presence of polybrene (8 *μ*g/mL) for 8 hours, and then cultured in fresh media for 48 hours. The infection efficiency was examined by the expression of GFP signals under fluorescent microscope and qPCR (Supplemental Figure 2). The ectopic expression of Mohawk was determined by qPCR and western blotting.

### 2.3. Growth Factor Treatment

BMSCs were seeded in 6- or 12-well plates at a density of 1 × 10^5^ cells/cm^2^ in BMSC growth medium for two days to reach about 90% confluence. For iPSCs, cells at passages 10-25 were split by 5 mM EDTA and seeded in 6- or 12-well plates at a density of 1 × 10^5^ cells/cm^2^ in BMSC basic medium for two days to reach about 90% confluence. Prior to *in vitro* tenogenic differentiation, cells were washed twice with BMSC basic medium, then treated with indicated concentrations of TGF-*β*3 or BMP12 (PeproTech, Inc., Rocky Hill, NJ) in the same medium for another five days with one medium change two days after the first treatment.

### 2.4. Sirius Red Staining [30]

After treatment with indicated growth factors, cells grown in 12-well plates were washed twice with PBS, fixed with 70% ethanol for 30 minutes at room temperature, then washed with distilled H2O for three times before incubation with 0.1% Sirius red in saturated aqueous solution of picric acid for one hour. To quantify the stained nodules, the stain was solubilized with 0.2 mL of 0.1% NaOH and absolute methanol (1 : 1 (vol/vol)) for 30 minutes at room temperature. Solubilized stain (0.06 mL) was transferred to wells of a 96-well plate, and absorbance was measured at 540 nm. Data are presented as mean ± SD, *n* = 3.

### 2.5. Mechanical Stretch

As previously described [28], to test the effects of mechanical force on tenogenic gene expression in teno-iPSCs and BMSCs, cells were seeded on vitronectin-coated poly(ɛ-caprolactone) (80 kDa; Sigma-Aldrich, St. Louis, MO) nanofibrous scaffolds for 3 days, and then subject to cyclic uniaxial sinusoidal force from a customized bioreactor. The device was programmed to approximate sinusoidal waveforms equating to 3% strain amplitude (0%–6% strain) at a frequency of 1 Hz for 18 hours. At the end of mechanical stretch, samples were lysed in TRIzol reagent for RNA extraction. Static controls were treated identically but with no cyclic mechanical loading.

### 2.6. RNA Extraction, cDNA Synthesis, and Quantitative Real-Time PCR (qRT-PCR)

Samples were lysed in TRIzol reagent (Invitrogen), and total RNA was extracted according to the manufacturer's instruction. One microgram of RNA was then treated with RQ1 RNase-free DNase and then used for cDNA synthesis by using a High-Capacity cDNA Reverse Transcription Kit (Thermo Fisher Scientific). Equine-specific primer pairs were designed using NCBI primer-blast or published data [18], and the list of primer sequences can be found in Supplemental Table 1. qPCR was carried out using SYBR Green PCR master mix (Biotool, USA) on an Applied Biosystems 7500 real-time PCR system. All PCRs were performed in triplicates. PCR cycle parameters were 95°C for 10 min, followed by 40 cycles of 95°C for 15 s, 60°C for 15 s, and 72°C for 15 s. At the end of the program, a melt curve was produced by taking readings every 1°C from 65 to 95°C. The reference gene PSMB2 was used to normalize gene expression, and relative fold changes were calculated using 2^−*ΔΔ*Ct^ method.

### 2.7. Western Blot

Cells were washed twice with PBS and then lysed in ice-cold T-PER buffer (Thermo Fisher Scientific) supplemented with protease inhibitor cocktail (Milipore Sigma). SDS-PAGE was carried out using the minigel system from Bio-Rad, and proteins were transferred to PVDF membranes. After blocking with TBST containing 5% nonfat dry milk for at least one hour at room temperature, the membranes were incubated at 4°C overnight with primary antibodies, followed by incubation with horseradish peroxidase-conjugated secondary antibodies for one hour at room temperature. After thorough washing with TBST buffer, signals on the membranes were developed with an enhanced chemiluminescent system (Pierce). Antibodies used in this study include the following: scleraxis (Abcepta #AP21316b, 1 : 1000), tenomodulin (Santa Cruz Technology #sc-49325, 1 : 1000), Mohawk (Abcam #ab179597, 1 : 1000), *α*-tubulin (Cell Signaling Technology #3873, 1 : 1000), p-SMAD3 (Santa Cruz Technology #sc-517575, 1 : 1000), and p-SMAD1/5 (Cell Signaling Technology #9516T, 1 : 1000).

### 2.8. Statistics

Data were presented as means ± STDEV. Statistical analysis was performed by ANOVA single-factor test in gene expression between the control and treated groups. A value of *p* < 0.05 was considered to be statistically significant.

## 3. Results

### 3.1. Dose Effects of TGF-*β*3 on Tenogenic Gene Expression in BMSCs and teno-iPSCs

**Figure 1:**
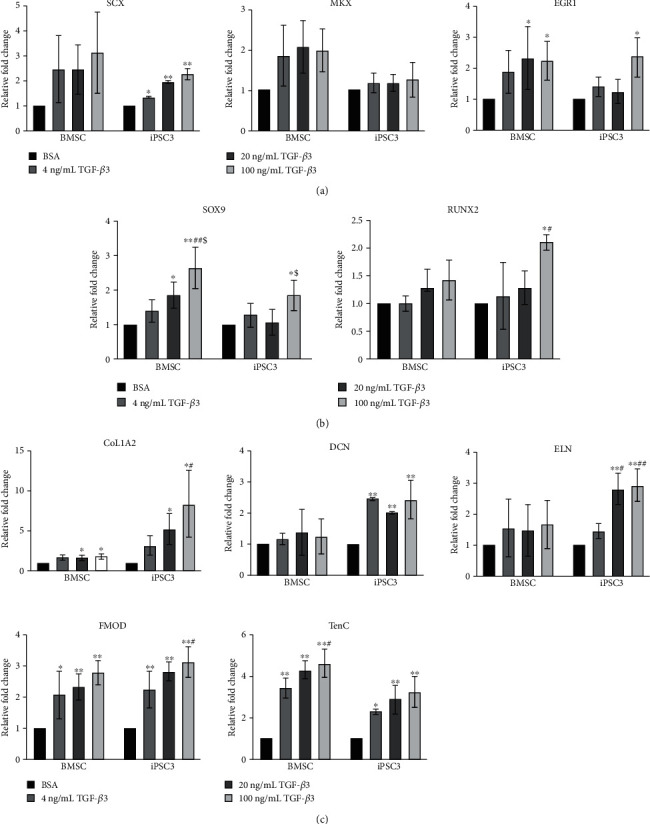
Dose effects of TGF-*β*3 on tenogenic gene expression in BMSCs and teno-iPSCs. Cells were treated with vehicle medium (0) or various concentrations of TGF-*β*3 (4, 20, and 100 ng/mL) for 5 days, and cDNA was synthesized from total RNA. Expression of tenogenic transcription factors (a), chondrogenic transcription factor SOX9, osteogenic transcription factor RUNX2 (b) and tenocyte-related ECM genes (c) was determined by qPCR. Relative fold change for each group was calculated by comparison to vehicle medium group, and data for BMSCs were summarized from 3 horses, and data for teno-iPSCs were summarized from 3 passages. ^∗^Data were compared to BSA control; ^#^data were compared to the 4 ng/mL group; ^$^data were compared to the 20 ng/mL group.

To evaluate the effects of TGF-*β*3 on tenogenic gene expression, BMSCs and teno-iPSC (clone3, iPSC3) were treated with three different concentrations of TGF-*β*3 for 5 days, and the expression of tenogenic transcription factors (*SCX*, *MKX*, and *EGR1*), chondrogenic master transcription factor *SOX9*, osteogenic master transcription factor *RUNX2*, and tendon-related ECM genes (*COL1A2*, *DCN*, *ELN*, *FMOD*, and *TNC*) was determined by qPCR. As shown in Figure 1, the levels of *FMOD* and *TNC* in BMSCs and the levels of *SCX*, *FMOD*, and *TNC* in iPSC3 were increased in a dose-dependent manner. The expression of *EGR1*, *SOX9*, and *COL1A2* in BMSCs tended to increase at low concentration of TGF-*β*3 (4 ng/mL) but were significantly upregulated at higher concentrations of TGF-*β*3 (20 ng/mL and 100 ng/mL). The expression of *SCX*, *MKX*, *RUNX2*, *DCN*, and *ELN* in BMSCs also trended upwards with the treatment. In iPSC3, TGF-*β*3 induced significant increase of *EGR1*, *SOX9*, *RUNX2*, *COL1A2*, and *ELN* at higher concentrations (20 ng/mL and/or 100 ng/mL) but not at low concentration. TGF-*β*3 also dramatically increased the expression of *DCN* at all three tested concentrations, but not in a dose-dependent manner (Figure 1(c)) within this range. Additionally, our previous work has reported that the retention of parental lineage genes varies among teno-iPSC clones and that iPSC3 displays higher levels of tenogenic gene expression than teno-iPSC clone 1 (iPSC1) does [28]. To compare the isogenic differentiation capacity between different iPSC clones, the response of iPSC1 to the tenogenic stimuli was also assessed in this study. As shown in Supplemental Figure 3, iPSC1 showed a similar pattern as iPSC3 on the expression of *SCX*, *SOX9*, and *COL1A2* with TGF-*β*3 treatment. Moreover, increase of *EGR1*, *RUNX2*, *DCN*, *ELN*, and *TNC* was detected at higher concentrations of TGF-*β*3. Taken together, these data indicate that the TGF-*β*3-activated tenocyte-related genes differ from individual cell types.

### 3.2. Dose Effects of BMP12 on Tenogenic Gene Expression in BMSCs and teno-iPSCs

**Figure 2:**
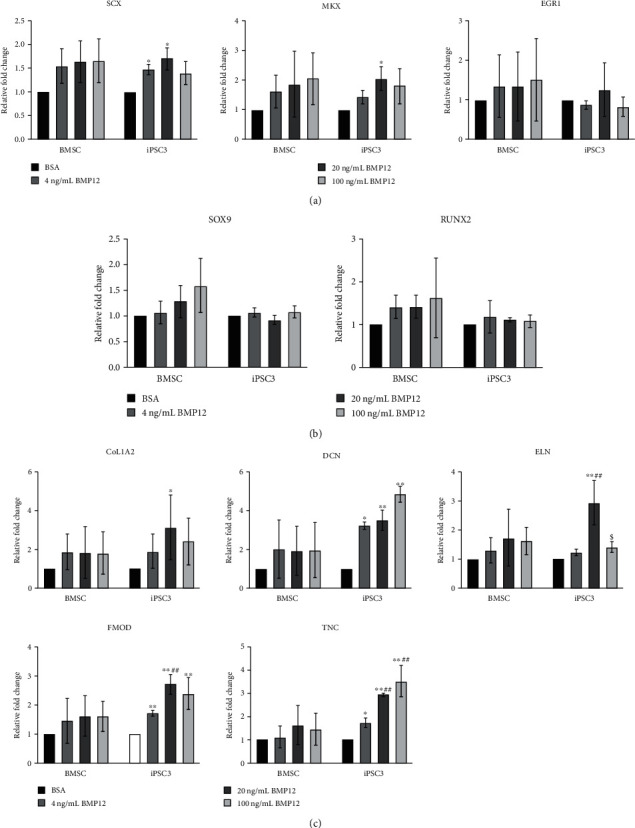
Dose effects of BMP12 on tenogenic gene expression in BMSCs and teno-iPSCs. Cells were treated with vehicle medium (0) or various concentrations of BMP12 (4, 20, and 100 ng/mL) for 5 days, and cDNA was synthesized from total RNA. Expression of tenogenic transcription factors (a), SOX9 and RUNX2 (b), and tenocyte-related ECM genes (c) was determined by qPCR. Relative fold change for each group was calculated by comparison to vehicle medium group. ^∗^Data were compared to BSA control; ^#^data were compared to the 4 ng/mL group; ^$^data were compared to the 20 ng/mL group.

To assess the effects of BMP12 on the tenogenic differentiation potential of BMSCs and teno-iPSCs, cells were treated with three different concentrations of BMP12 for 5 days, and gene expression was measured by qPCR. As shown in Figure 2, the expression of *SCX*, *MKX*, *EGR1*, *SOX9*, *RUNX2*, *COL1A2*, *DCN*, *ELN*, *FMOD*, and *TNC* tended to increase at all three tested concentrations in BMSCs. On the other hand, BMP12 treatment increased the expression of *DCN* and *TNC* in a dose-dependent manner in iPSC3, where the expression of *SCX*, *MKX*, *COL1A2*, and *ELN* was upregulated by BMP12 at higher concentrations (20 ng/mL and/or 100 ng/mL). As to iPSC1, while the expression of *SCX*, *MKX*, *EGR1*, *DCN*, and *RUNX2* trended upwards, BMP12 significantly increased the expression of *SOX9*, *COL1A2*, and *ELN* at all three concentrations (Supplemental Figure 4). Collectively, these data suggest that, similar to TGF-*β*3, the BMP12-induced tenocyte-related genes are also varied between cell types.

### 3.3. Effects of TGF-*β*3 and BMP12 on Cell Morphology and Tenogenic Protein Expression in BMSCs and teno-iPSCs

**Figure 3:**
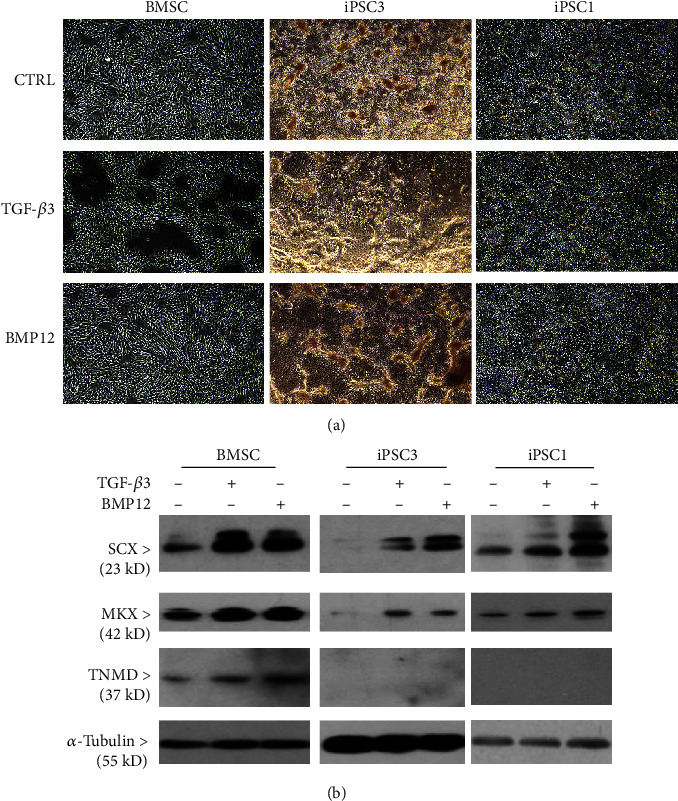
Effects TGF-*β*3 and BMP12 on cell morphology and tenogenic protein expression. BMSCs and tow teno-iPSC clones (iPSC3 and iPSC1) were treated with vehicle medium, TGF-*β*3 (20 ng/mL), or BMP12 (20 ng/mL) for 5 days. The cell morphology was imaged (a), and whole cell lysates were blotted for scleraxis (SCX), Mohawk (MKX), tenomodulin (TNMD), and *α*-tubulin (b).

As mentioned above, TGF-*β*3 and BMP12 stimulated the expression of many tenogenesis-related genes at the transcriptional level in BMSCs and teno-iPSCs. The change of intrinsic molecular content may be indicated by alterations in cell morphology. Because the highest concentration (100 ng/mL) greatly increased the expression of *SOX9* and *RUNX2*, growth factors at 20 ng/mL were used for further experiments. Compared to cells treated with BSA vehicle medium, BMSCs and iPSC3 were more inclined to form clusters after treatment with TGF-*β*3 for 5 days, which was less evident in iPSC1 (Figure 3(a)). Morphology differences were discernible in all the tested cells when they were exposed to BMP12.

To determine the effects of TGF-*β*3 and BMP12 on the expression of tenogenic proteins in BMSCs and teno-iPSCs, cells were treated with TGF-*β*3 or BMP12 for 5 days, whole cell lysates were immunoblotted with antibodies against SCX and MKX. As shown in Figure 3(b), both TGF-*β*3 and BMP12 apparently enhanced the expression of SCX and MKX in BMSCs and two teno-iPSC clones. Tenomodulin is believed to be a marker for mature tenocytes. We failed to measure TNMD gene expression by RT-PCR (data not shown); however, immunoblotting with antibodies against TNMD protein showed specific signals at expected size for cell lysates from BMSCs, and the signals were greatly enhanced by TGF-*β*3 and BMP12 stimulation. Surprisingly, no TNMD signals were detected in the two teno-iPSC clones with either treatment (Figure 3(b)).

Additionally, to evaluate the effects of TGF-*β*3 and BMP12 on collagen deposition, treated cells were stained with Sirius red. As shown in Supplemental Figure 5, the intensity of Sirius red staining was significantly increased by TGF-*β*3 in BMSCs. Quantification data also showed a slight but significant increase of staining in TGF-*β*3-treated iPSC3 and iPSC1. This effect was not significant with BMP12 treatment in either types of cells.

### 3.4. Effects of TGF-*β*3 and BMP12 on Tenogenic Gene Expression in MKX-Overexpressing BMSCs and teno-iPSCs

**Figure 4:**
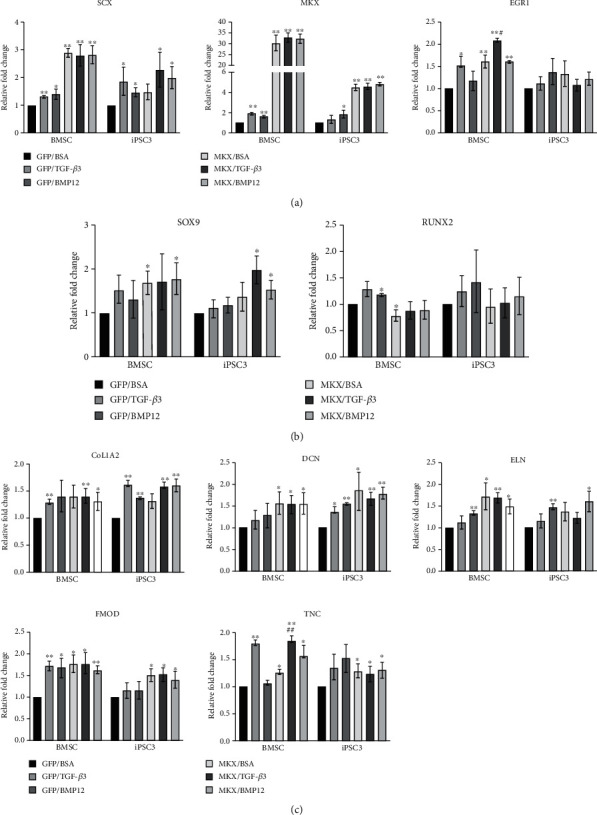
Effects of TGF-*β*3 or BMP12 on tenogenic gene expression in MKX-overexpressing BMSCs and teno-iPSCs. Cells expressing GFP or equine Mohawk were treated with vehicle medium (GFP/BSA and MKX/BSA), TGF-*β*3 (20 ng/mL, GFP/TGF-*β*3 and MKX/TGF-*β*3), or BMP12 (20 ng/mL, GFP/BMP12 and MKX/BMP12) for 5 days. cDNA was synthesized from total RNA, and expression of tenogenic transcription factors (a), SOX9 and RUNX2 (b), and tenocyte-related ECM genes (c) was determined by qPCR. Relative fold change for each group was calculated by comparison to the GFP-CTRL group. ^∗^Data were compared to the GFP/BSA group, and ^#^data were compared to the MKX/BSA group.

Our previous work has shown that ectopic expression of Mohawk stimulates the tenogenic gene expression in both BMSCs and teno-iPSCs [28]. In line with this notion, compared to control GFP-expressing cells, overexpression of MKX increased the expression of *SCX*, *EGR1*, *SOX9*, *DCN*, *ELN*, *FMOD*, and *TNC* in BMSCs (MKX-BMSCs) and *COL1A2*, *DCN*, *FMOD*, and *TNC* in iPSC3 (MKX-iPSC3, Figure 4 and Supplemental Figure 6) and iPSC1 (MKX-iPSC1, Supplemental Figure 6 & 7). To determine the synergistic effects of forced expression of MKX with TGF-*β*3 or BMP12 on tenogenic gene expression, GFP- or MKX-expressing cells were exposed to TGF-*β*3 or BMP12 for 5 days, and the gene expression was measured by qPCR. As shown in Figure 4, TGF-*β*3 treatment further enhanced the expression of *EGR1* and *TNC* in MKX-BMSCs and trended to further increase the expression of *SCX*, *SOX9*, *COL1A2*, and *FMOD* in MKX-iPSC3 and *SCX*, *SOX9*, *RUNX2*, *COL1A2*, and *DCN* in MKX-iPSC1. On the other hand, BMP12 treatment trended to increase the expression of *TNC* in MKX-BMSCs, *SCX*, *RUNX2*, *SOX9*, and *COL1A2* in MKX-iPSC3 and *RUNX2*, *COL1A2*, *DCN*, and *TNC* in MKX-iPSC1 (Supplemental Figure 7).

### 3.5. Effects of Mechanical Stretch on Tenogenic Gene Expression in TGF-*β*3- and BMP12-Treated BMSCs and teno-iPSCs

**Figure 5:**
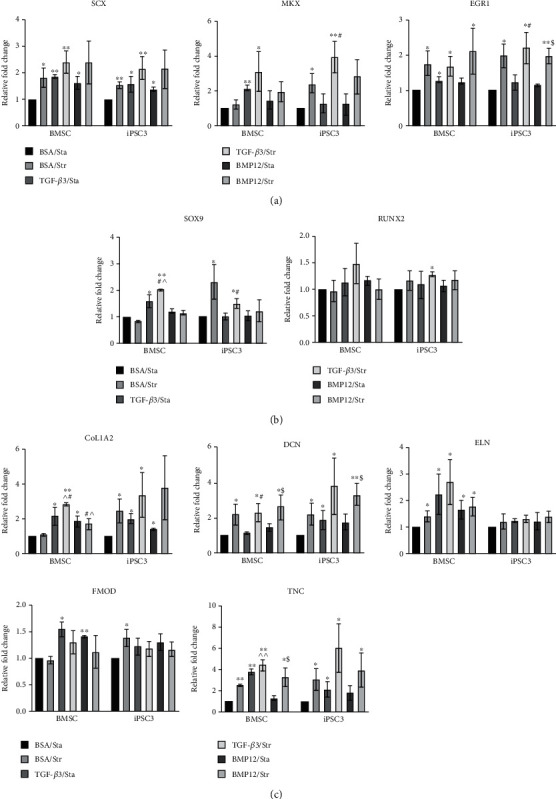
Effects of cyclic uniaxial mechanical stretch on gene expression in TGF-*β*3- or BMP12- treated BMSCs and teno-iPSCs. BMSCs and teno-iPSCs were seeded on vitronectin-coated PCL scaffolds for 2 days in basic medium, and then treated with vehicle medium, TGF-*β*3, or BMP12 for 2 days prior to uniaxial mechanical stretch for 18 hours in the presence of vehicle medium (BSA/Str), TGF-*β*3 (TGF-*β*3/Str), or BMP12 (BMP12/Str). Cells seeded on PCL scaffolds without mechanical loading but with vehicle medium (BSA/Sta), TGF-*β*3 (TGF-*β*3/Sta), or BMP12 (BMP12-Sta) were served as static control. Expression of tenogenic transcription factors (a), SOX9 and RUNX2 (b), and tenocyte-related ECM genes (c) was determined by qPCR, and relative fold change for each group was calculated by comparison to BSA/Sta group. ^∗^Data were compared to the BSA control group; ^data were compared to BSA/Str; ^$^data were compared to BMP12/Sta.

Both molecular cues and mechanical loading play essential roles in tendon development and homeostasis. Our previous study has reported that mechanical stretch affects tenogenic gene expression in BMSCs and teno-iPSCs [28]. In accordance with this statement, compared to static condition, cyclic uniaxial stretch increased the expression *SCX*, *EGR1*, *DCN*, *ELN*, and *TNC* in BMSCs and *SCX*, *MKX*, *EGR1*, *SOX9*, *COL1A2*, *DCN*, *FMOD*, and *TNC* in iPSC3. To determine the synergistic effects of mechanical stretch with TGF-*β*3 or BMP12 on tenogenic gene expression, cells were pretreated with TGF-*β*3 or BMP12 prior to cyclic uniaxial mechanical tensile, and the expression of tenocyte-related genes was determined by qPCR. As shown in Figure 5, TGF-*β*3 increased the expression of *SCX*, *MKX*, *EGR1*, *SOX9*, *COL1A2*, *FMOD*, *ELN*, and *TNC* in static BMSCs and *SCX*, *COL1A2*, *DCN*, and *TNC* in static iPSC3. Exposure of TGF-*β*3 pretreated cells to mechanical stretch increased the expression of *SCX*, *MKX*, *SOX9*, *COL1A2*, *ELN*, and *TNC* in BMSCs and *SCX*, *MKX*, *RUNX2*, *COL1A2*, *DCN*, and *TNC* in iPSC3. On the other hand, BMP12 treatment elevated the expression of *SCX*, *COL1A2*, *ELN*, and *FMOD* in static BMSCs and *SCX* and *COL1A2* in static iPSC3. Mechanical loading on BMP12-pretreated cells upregulated the levels of *EGR1*, *DCN*, and *TNC* in BMSCs and *EGR1* and *DCN* in iPSC3. Taken together, these data indicate that mechanical stretch and growth factors synergistically regulate tenogenic gene expression in a cell type-dependent manner.

### 3.6. Potential Signaling Networks Associated with Tenogenic Gene Expression in BMSCs and teno-iPSCs

**Figure 6:**
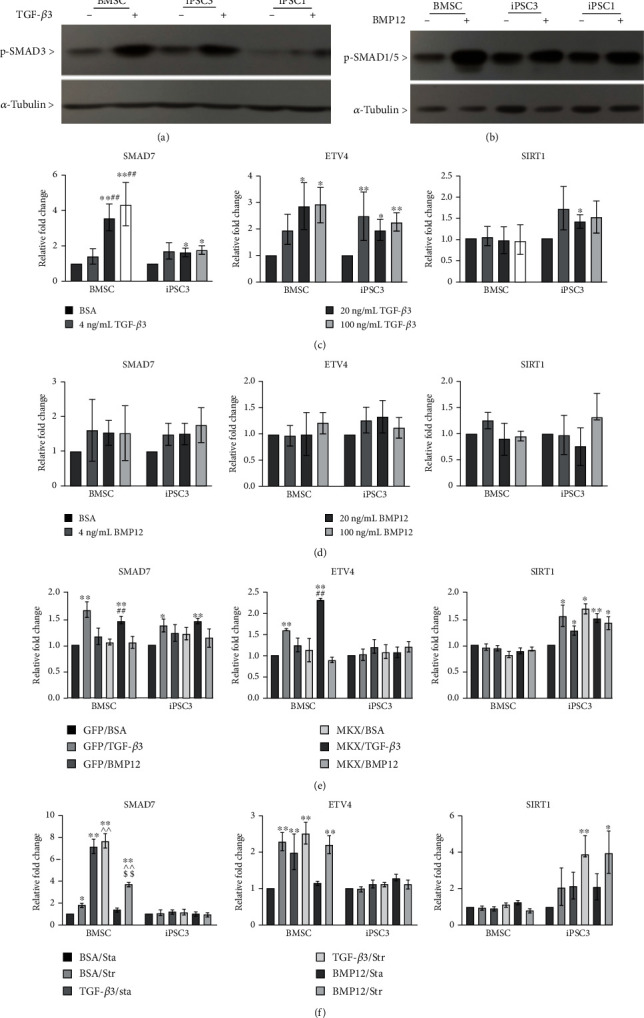
Effects of tenogenic stimuli on the expression of signaling factors. (a) BMSCs and teno-iPSC were treated with vehicle medium or TGF-*β*3 (20 ng/mL) for 1 hr, and whole cell lysates were blotted for phosphorylated SMAD3 and a-tubulin. (b) BMSCs and teno-iPSCs were treated with vehicle medium or BMP12 (20 ng/mL) for 1 hr, and whole cell lysates were blotted for phosphorylated SMAD3 (p-SMAD3) and a-tubulin. (c) Cells were treated as in Figure 1, and the expression of *SMAD7*, *ETV4*, and *SIRT1* was determined by qPCR. ^∗^Data were compared to BSA control; ^#^data were compared to the 4 ng/mL group. (d) Cells were treated as in Figure 2, and the expression of *SMAD7*, *ETV4*, and *SIRT1* was determined by qPCR. (e) Cells were treated as in Figure 4, and the expression of *SMAD7*, *ETV4*, and *SIRT1* was determined by qPCR. ^∗^Data were compared to the GFP/BSA group, and ^#^data were compared to the MKX/BSA group. (f) Cells were treated as in Figure 5, and the expression of *SMAD7*, *ETV4*, and *SIRT1* was determined by qPCR. ^∗^Data were compared to the BSA control group; ^data were compared to BSA/Str; ^$^data were compared to BMP12/Sta.

TGF-*β* ligands phosphorylate and activate the receptor-regulated transcription factors SMAD2/3 or SMAD1/5/8 via binding to transmembrane TGF-*β* receptors [31]. As expected, in all tested cells TGF-*β*3 and BMP12 greatly enhanced the phosphorylated form of SMAD3, and SMAD1/5, respectively (Figures 6(a) and 6(b)). It has also been acknowledged that the TGF-*β* superfamily regulates cell proliferation and differentiation through not only the canonical SMAD signaling but also the SMAD-independent noncanonical pathways [32]. In line with this notion, the mRNA levels of *SMAD7*, one inhibitory Smad that negatively controls both TGF-*β* and BMP-induced SMAD signaling [33], were significantly increased by TGF-*β*3 at higher concentrations in BMSCs and teno-iPSCs (Figure 6(c)). These phenomena were not observed when cells were treated with BMP12 or overexpressing MKX (Figures 6(d) and 6(e)). Interestingly, mechanical stretch resulted in an apparent increase of *SMAD7* expression in BMSCs, but not in iPSCs (Figure 6(f)), suggesting that regulation of TGF-*β* signaling by mechanical force might be cell type dependent. In addition, the expression of *ETV4*, a gene that can be used as transcriptional readout of ERK/MAPK activity [34], was highly upregulated by TGF-*β*3 in BMSCs and iPSC3 but not in iPSC1 (Figure 6(c), Supplemental Figure 1). Meanwhile, activation of *ETV4* was also revealed in BMSCs by mechanical force (Figure 6(f)), but not in cells treated by either BMP12 or ectopic expression of MKX (Figures 6(d) and 6(e), Supplemental Figures 2 and 4), implying that activation of ERK/MAPK signaling is dependent on cell type as well as on tenogenic inducers. On the other hand, to understand whether an epigenetic modifier was affected by tenogenic inducers, the transcriptional activities of sirtuin-1 (*SIRT1*), one of the NAD-dependent histone deacetylases (HDACs), were determined by qPCR on stimulated cells. The results showed that *SIRT1* expression in BMSCs and iPSC1 was barely affected by any of the tested stimuli. However, its level in iPSC3 was slightly but significantly elevated by TGF-*β*3 at 20 ng/mL, overexpression of MKX alone or combined with TGF-*β*3 or BMP12, and mechanical stretch combined with TGF-*β*3 or BMP12 (Figure 6 and Supplemental Figure 4), suggesting that activation of *SIRT1* gene by tenogenic stimuli is dependent on intracellular context.

## 4. Discussion

In this study, we extensively examined the effects of individual or combined tenogenic cues, including TGF-*β*3, BMP12, ectopic expression of MKX, and mechanical stretch, on the expression of tenocyte-related genes in teno-iPSCs and BMSCs. Our data revealed that those stimuli affected the activities of tenogenic transcription factors, including *SCX*, *MKX*, and *EGR1*, and the expression of tendon-related ECM genes, such as *COL1A2*, *DCN*, *ELN*, *FMOD*, and *TNC*. Moreover, those tenogenic inducers also showed high impact on the expression of signaling regulators *SMAD7*, *ETV4*, and *SIRT1* in BMSCs and teno-iPSCs.

### 4.1. Regulation on Tenocyte-Associated Transcription Factors

Although the exact mechanisms triggering tenogenesis still remain elusive, to date, at least three transcription factors, i.e., SCX, MKX, and *EGR1*, have been reported to play essential roles in tendon development. Depletion of either genes caused apparent tendon abnormalities [35–38]. In other words, stimulation of those genes may drive the stem cell fate to tenocyte lineage. As the first transcription factor found to be required for tendon formation, SCX is a widely accepted tenogenic marker in *in vitro* studies. While it is still not fully understood how *SCX* activity is regulated *in vivo*, the loss of SCX signals in TGF-*β*2^−/−^- and TGF-*β*3^−/−^-deficient mouse embryos suggest that TGF-*β* signaling is needed for *SCX* expression in developing tendon [13]. In our study, treatment with TGF-*β*3 or BMP12 greatly increased *SCX* expression at the protein level in both teno-iPSCs and BMSCs, suggesting TGF-*β* ligands may be served as a potent tenogenic inducer to program stem cells towards tenocytes. Moreover, our study also showed that cyclic mechanical loading alone (1.0 Hz with 0%-6% sinusoidal wave of strain for 18 hrs) was able to enhance the expression of *SCX* in both BMSCs and teno-iPSCs. This is in line with the notion that mechanical stress is an inducer of *SCX* expression [39], although another study from Brown et al. reported that it was not mechanical stress alone (1 hr/day of 0.5 Hz with 1% strain for 3 days), but TGF-*β*2 or TGF-*β*2 combined with mechanical stress that increased *SCX* expression in mouse BMSCs [40]. This discrepancy may be due to the different stretch parameters applied. Nevertheless, an earlier study from Maeda et al. showed that physical forces could regulate the release of active TGF-*β* from ECM, thus fine-tune *SCX* expression through TGF-*β*/SMAD2/3-mediated signaling [41]. This might also be the reason for the synergistic effects of TGF-*β*3 and mechanical loading on *SCX* expression in both BMSCs and teno-iPSCs. Additionally, our data also demonstrated that *SCX* expression could be promoted by forced expression of *MKX*, especially in BMSCs. This is in agreement with the report that ectopic expression of MKX dramatically increased the level of *SCX* in C3H10T1/2 cells through TGF-*β* signaling [42], but not in accord with other studies where MKX did not activate the expression of *SCX* in human BMSCs [27], or in mouse periodontal ligament (PDL) fibroblasts [43] or Achilles tendons [44]. This disparity suggests that the capability of MKX to regulate *SCX* or other targets may differ between species and cell types.

On the other hand, while MKX is highly expressed in developing tendons and plays important roles in tenogenic differentiation, there is very limited information on its upstream regulator(s) [45]. BMP12 has been reported to be one of the growth factors able to activate *Mkx* in a variety of mesenchymal stem cells [27, 46, 47]. In our study, this effect was not evident with qPCR analysis; however, results from western blotting showed apparent higher levels of MKX in BMP12- or TGF-*β*3-treated BMSCs and teno-iPSCs than those in vehicle controls, implying a role of TGF-*β* signaling in the control of *MKX* expression. Furthermore, *MKX* activation can also be induced by mechanical loading as it was greatly elevated in rat patellar tendon-derived cells upon exposure to mechanical tensile (4% monoaxial cyclic elongation for 6 hrs) [37]. An *in vivo* study from Kayama et al. also showed increased level of *MKX* in treadmill mouse Achilles tendon [45]. The authors further reported that mechanical stretch (0.25 Hz with 2% strain for 6 hrs) induced the nuclear translocation of transcription factor Gtf2ird1 in rat primary Achilles tenocytes, thus boosted the expression of *MKX*. In our study, with respect to *MKX* expression, mechanical loading showed more significant effects in teno-iPSCs than that in BMSCs. It will be of great interest to know whether *Gtf2ird* also mediates the biomechanical responses in those cells.

In addition to SCX and MKX, the zinc finger transcription factor *EGR1* also appears to play important roles in controlling tendon development, homeostasis, and repair [29, 38, 48]. It is known that *EGR1* can be induced in various tissues by multiple extracellular stimuli, such as growth factors and mechanical signals. However, it remains unclear how *EGR1* is regulated by biochemical cues during tendon formation and *in vitro* tenogenic differentiation. Guo et al. reported that *EGR1* level was highly enhanced in rat TPSCs treated with 10 ng/mL TGF-*β*1 for 10 days [5], but the study from Yin et al. showed decreased expression of *EGR1* in rat BMSCs treated with the same concentration of TGF-*β*1 for 3 or 7 days [30]. Another study from Guerquin et al. showed no changes on *EGR1* expression in C3H10T1/2 cells treated with 20 ng/mL TGF-*β*2 for 1 or 24 hrs [38]. In our study, enhanced expression of *EGR1* was observed in equine BMSCs treated with TGF-*β*3 at 20 ng/mL or 100 ng/mL and in teno-iPSCs treated with 100 ng/mL TGF-*β*3. These data suggest that induction of *EGR1* by TGF-*β* may be cell type and concentration dependent. Additionally, BMP12 was also reported to be able to induce *EGR1* expression in turkey BMSCs [49]. However, in our study, BMP12 only tended to increase *EGR1* in teno-iPSC1 but not in BMSCs and teno-iPSC3. These results are partially in line with the findings from the other study where *EGR1* expression in rat BMSCs was not influenced by BMP12 [30]. Of note, as *EGR1* is a well-known mechanosensitive gene, it is expected to observe apparent increase of *EGR1* in BMSCs and teno-iPSCs upon mechanical loading, which may override the effects resulted from another stimulus.

It is also worth noting that the tenogenic stimuli used in our study influenced the activities of chondrolineage-related transcription factor *SOX9* and osteolineage-related transcription factor *RUNX2*. For example, the level of *SOX9* in BMSCs was dose dependently upregulated by TGF-*β*3 and trended upwards by BMP12, while in teno-iPSC3, it was elevated by a high dose of TGF-*β*3 and/or mechanical stretch. Moreover, the expression of *RUNX2* was decreased in BMSCs and teno-iPSC1 by ectopic expression of MKX, but increased in teno-iPSCs by a high dose of TGF-*β*3. These results are not surprising because TGF-*β* signaling, mechanical loading, and MKX are also known to play important roles in regulating cartilage and bone formation [37, 50]. Nevertheless, our results indicate that forced expression of MKX may attenuate the risk of bone formation in tendon repair with certain types of stem cells that are preprogrammed by growth factors.

### 4.2. Regulation on Tendon-Related Extracellular Matrix Gene Activity

Precisely organized tendon matrix is synthesized by tendon cells and predominantly composed of type I collagen, together with small amount of other types of collagens and noncollagenous materials [51]. Tendon injury is usually associated with disrupted structures, and the repair/healing process is involved in rebuilding the injured tissue back with normal functions. Hence, although many of them are not tendon specific, the expression of ECM-related genes is often used as reference to evaluate the potential of stem cell therapy for tendon disorders. In this study, we determined the levels of ColI subunit *COL1A2*, decorin (*DCN*), elastin (*ELN*), fibromodulin (*FMOD*), and tenascin-c (*TNC*) in stimulated BMSCs and teno-iPSCs. Our data revealed that *COL1A2* was upregulated in all the tested cells by treatment involved with TGF-*β*3. This is consistent with the fact that TGF-*β* stimulates the binding of ubiquitous transcription factor Sp1, the SMAD3/4 complex, and the coactivators p300/CBP to *COL1A2* promoter [52]. Moreover, in teno-iPSCs, *COL1A2* was also activated by overexpression of MKX, BMP12/MKX, or mechanical stretch. Since the expression of MKX in teno-iPSCs was enhanced by mechanical loading, it is reasonable to presume that the activity of *ColA2* in teno-iPSCs can be regulated by MKX. Indeed, previous studies have shown that *COL1A2* level was decreased in *MKX*^−/−^ mice [43, 44] and increased in MKX-overexpressing PDL fibroblasts [43]. Our data also demonstrated that overexpression of MKX alone or combined with TGF-*β*3 or BMP12 increased or trended to increase the expression of *COL1A2*, *DCN*, *ELN*, *FMOD*, and *TNC* in all the tested cells, further supporting that MKX plays crucial roles in regulating ECM gene activities in BMSCs and teno-iPSCs.

Decorin (DCN), the most abundant noncollagenous matrix protein in the tendon [53], participates in collagen fibril organization and prevents fibrosis formation [54]. In the present study, *DCN* level was increased in both BMSCs and teno-iPSCs by mechanical tensile-related inducer. This is partially in agreement with the study from Youngstrom et al. but contradictory to other studies where mechanical stimulation decreased *DCN* expression in human primary rotator cuff fibroblasts and C3H10T1/2 cell lines [55–57]. Another study from Chen et al. showed no changes on *DCN* level when human ES-derived MSCs were subject to mechanical stress for 24 hrs [26]. These inconsistencies may be due to different stretch parameters applied. Indeed, Xu et al. reported that *DCN* expression was increased by moderate treadmill running but decreased by strong treadmill running in rat Achilles tendon [58]. Of note, our results also revealed that treatment with TGF-*β*3 or BMP12 resulted in a significant elevation of *DCN* in teno-iPSCs but not in BMSCs, suggesting that regulation of *DCN* activity by TGF-*β* signaling is cell type dependent.

Fibromodulin is reported to be essential for the maintenance of tendon stem cell niches [3], and its deficiency resulted in a structurally and mechanically abnormal tendon phenotype [59]. Xu et al. reported that cyclic tensile strain induced the expression of *FMOD* in rat TPSCs [60]; however, our data demonstrated that mechanical loading showed little effects on *FMOD* activity in BMSCs and teno-iPSCs, suggesting that regulation of FMOD expression by mechanical force also varies on cell type. In addition, the study from Tan et al. showed that targeted deletion of TGF-*β*r2 decreased the level of *FMOD* in mouse tenocytes [15], suggesting that TGF-*β* signaling is involved in *FMOD* activity. Indeed, in our study, its level was upregulated in TGF-*β*3-treated BMSCs and in TGF-*β*3- or BMP12-treated teno-iPSCs.

Elastin is the core protein of elastic fibers with unique ability to sustain large deformation [61]. While disrupted elastic fibers are associated with the development of chronic tendinopathy [62], increased expression of elastin in injured tendons suggests that it may play a role in the healing process [38, 63]. In *MKX*^−/−^ mouse Achilles tendon, *ELN* level was much higher than that in the wildtype, indicating MKX may repress *ELN* gene activity [44]. Our data, however, is somewhat contradictory to that finding as overexpression of MKX elevated the level of *ELN* in BMSCs but not in teno-iPSCs, suggesting that the target(s) of transcription factor MKX is cell type dependent. In addition, whilst Min et a.l reported that mechanical strain downregulated the expression of *ELN* in human parametrial ligament fibroblasts [64], our results demonstrated that mechanical tensile-related inducers upregulated *ELN* in BMSCs. This inconsistency implied that the response of *ELN* gene to biophysical force may also rely on the cell type.

Tenascin C is expressed relatively low in mature tendon and suggested to play a role in proper alignment and orientation of collagen fibrils within the tendon [65]. Significant increase of *TNC* in acutely injured equine tendon indicates that it may also contribute to tendon repair [66]. Previous studies have shown that *TNC* activity can be affected by both biochemical and biomechanical cues [67]. In our study, all the tested stimuli enhanced the expression of *TNC* in teno-iPSCs, and similar results were obtained from BMSCs treated with all stimuli except BMP12. Although these data did not agree with the studies showing decreased *TNC* in mechanical stressed C3H10T1/2 cells [57], increased *TNC* in BMP12-treated rat BMSCs [46], and variable expression of *TNC* in TGF-*β*3-treated equine ESCs and iPSCs [18], they are in agreement with other studies reporting increased *TNC* in mechanical strained human BMSCs [68] and in TGF-*β*3-treated equine ADMSCs [69].

Tenomodulin, one of the transmembrane glycoproteins, has been widely accepted as a specific marker for tenogenic differentiation because it is predominantly expressed in tendon and ligament. SCX is so far the only transcription factor found to directly transactivate *TNMD* via E-boxes to positively regulate tenocyte differentiation and maturation [70]. In our study, although for unknown reason, qPCR with several sets of primers failed to detect *TNMD* at the RNA level in any type of cells used in this study, results from western blotting revealed apparent increases of SCX and TNMD in TGF-*β*3- or BMP12- treated BMSCs. On the other hand, Kayama et al. showed that deletion of *MKX* upregulated the expression of *SCX* but not that of *TNMD* in mouse Achilles tendon [45], implying that activation of *SCX* does not always correlate with the expression of *TNMD*. In the current study, in spite of evident expression of SCX, no TNMD was detected in either control or stimulated teno-iPSCs, suggesting a cofactor(s), which is likely not expressed or insufficient in teno-iPSCs, might be required for SCX-mediated *TNMD* activation.

Taken together, our results demonstrated that activation of tenogenic genes was not only dependent on the inducers but also varied between cell types. In respect to the expression of mature tenocyte marker TNMD and to the reduction of osteogenic gene expression, activation of TGF-*β* signaling by TGF-*β*3 or BMP12 combined with ectopic expression of transcription factor Mohawk may be suitable for BMSCs towards the tenocyte-lineage differentiation. However, the lack of TNMD expression in teno-iPSCs requires further work to optimize the condition for their tenogenic differentiation.

### 4.3. Regulation on Potential Signaling Factors

The molecular mechanisms underlying tendon development are generally thought to play similar roles in adult tissue regeneration. Upon injury, a variety of growth factors and cytokines are released from the injured tendons and adjacent tissues [71], and different signaling pathways, including TGF-*β*-SMAD2/3, BMP-SMAD1/5/8, ERK/MAPK, mTOR, and Wnt/*β*-catenin, are reported to associate with tendon development and repair [34, 72–74]. *SMAD7* is known to be a TGF-*β*-inducible antagonist of TGF-*β* signaling [33]. It can also be induced by other cytokines and growth factors, such as interferon-*γ*, tumor necrosis factor-*α*, and epidermal growth factor [75–77], suggesting that *SMAD7* is linked in crosstalk between divergent signaling pathways. In the present study, TGF-*β*3 stimulated the expression of *SMAD7* in both BMSCs and teno-iPSCs, implying a modulatory role of *SMAD7* in the negative feedback loop. Moreover, since mechanical loading is known to positively regulate TGF-*β* signaling, it is therefore not surprising to see increased level of *SMAD7* in stretched BMSCs. Interestingly, this effect was not observed in teno-iPSCs. One possible reason is that the biochemical signals converted from the mechanical force used in this study are inadequate to stimulate/maintain the expression of *SMAD7* in teno-iPSCs.

*ETV4* is a member of the ETS domain transcription factor family. Its transactivation capacity is enhanced following activation of the ERK- and JNK- MAPK signaling pathways [78], thus can be served as transcriptional readout of ERK/MAPK activity. In the current study, *ETV4* was upregulated by TGF-*β*3 in both BMSCs and iPSC3, indicating the intracellular crosstalk between the ERK and TGF*β* signaling pathways. Moreover, mechanical stretch has been shown to induce ERK1/2 phosphorylation in primary tendon fibroblasts [79], and our data also revealed an increase of *ETV4* expression in mechanical loaded BMSCs, suggesting that the ERK signaling can be activated by mechanical force. On the other hand, similar to the finding that ERK1/2 was not activated by mechanical force in human dermal keratinocyte cells [80], stretch-induced expression of *ETV4* was not evident in teno-iPSCs, implying that the mechanical loading-mediated ERK activation may be cell type dependent. Further work will be required to understand the roles of ERK signaling pathway in stem cell-based tenogenic differentiation.

Sirtuin-1 (*SIRT1*) is an NAD^+^-dependent class III HDAC targeting both histone and nonhistone proteins. It has been shown to inhibit the apoptosis and inflammatory response in human tenocytes [81] and to mediate the activation of immune/defense genes induced by mechanical stretch in human PDL cells [82]. Interestingly, while the class I/II HDAC inhibitors trichostatin A and valproic acid promoted SCX expression in mouse TSPCs [83], overexpression of *SIRT1* also upregulated *SCX* in rat BMSCs where the *SIRT1*-JNK/SMAD1-PPARg signaling pathway was accounted for BMP14-induced tenogenic differentiation [84]. Moreover, *SIRT1* was downregulated by TGF-*β* and identified as a crucial regulator of TGF-*β*/SMAD signaling in fibroblast activation and tissue fibrosis [85]. In our study, *SIRT1* in BMSCs and teno-iPSC1 was not influenced by any tested tenogenic stimulus, but it was enhanced in teno-iPSC3 under certain conditions, including overexpression of Mohawk, mechanical stretch combined with TGF-*β*3 or BMP12, indicating that the regulatory network of *SIRT1* gene activity is different among cell types. More studies will be needed to unveil the role of HDACs in the regulation of tenogenic gene expression in stem cells.

## 5. Conclusions

In summary, our results highlight that both BMSCs and teno-iPSC hold significant tenogenic differentiation capacity. However, the activation of tenogenic genes is highly dependent on the inducers and varies between iPSC clones as well as between cell types. Therefore, additional assessment on the expression of tenocyte-related genes will be needed to achieve the purpose of using predifferentiated stem cells for tendon repair and regeneration.

## Data Availability

All data used to support the findings of this study are included within the article.

## Supplementary Materials

Supplementary 1 Supplemental Figure 1 Multilineage differentiation capacity of BMSCs. A, B *In vitro* osteogenic differentiation of BMSCs. The calcium deposition was revealed by Alizarin Red S staining. C, D *In vitro* adipogenic differentiation of BMSCs. The fat droplets were displayed by oil-red staining. E, F *In vitro* chondrogenic differentiation of teno-iPSCs. The production of proteoglycan proteins was shown by Alcian blue staining.

Supplementary 2 Supplemental Figure 2 Expression of GFP in lentiviral-infected teno-iPSCs and BMSCs. Teno-iPSCs (A) and BMSCs (B) were infected with lentivirus expressing GFP alone or MKX and GFP for 5 days. GFP signal was imaged under fluorescent microscope.

Supplementary 3 Supplemental Figure 3 Dose effects of TGF-*β*3 on tenogenic gene expression in iPSC1. iPSC1 were treated with vehicle medium (0) or various concentrations of TGF-*β*3 (4, 20, and 100 ng/mL) for 5 days, and cDNA was synthesized from total RNA. Expression of tenogenic transcription factors (A), chondrogenic transcription factor Sox9, osteogenic transcription factor RUNX2 (B), and tenocyte-related ECM genes (C) was determined by qPCR. Relative fold change for each group was calculated by comparison to vehicle medium group, and data were summarized from 3 passages. ^∗^Data were compared to BSA control; ^#^data were compared to the 4 ng/mL group; ^$^data were compared to 20 ng/mL group.

Supplementary 4 Supplemental Figure 4 Dose effects of BMP12 on tenogenic gene expression in iPSC1. Cells were treated with vehicle medium (0) or various concentrations of BMP12 (4, 20, and 100 ng/mL) for 5 days, and cDNA was synthesized from total RNA. Expression of tenogenic transcription factors (A), SOX9, RUNX2 (B), and tenocyte-related ECM genes (C) was determined by qPCR. Relative fold change for each group was calculated by comparison to the vehicle medium group, and data were summarized from 3 passages. ^∗^Data were compared to BSA control; ^#^data were compared to the 4 ng/mL group; ^$^data were compared to the 20 ng/mL group.

Supplementary 5 Supplemental Figure 5 Sirius red staining in TGF-*β*3- and BMP12-treated teno-iPSCs and BMSCs. A Cells were treated with TGF-*β*3 and BMP12 for 5 days, then fixed and stained with Sirius red. B Quantitation of Sirius red staining. ^∗^*p* < 0.05; ^∗∗^*p* < 0.01.

Supplementary 6 Supplemental Figure 6 Overexpression of MKX in BMSCs and teno-iPSCs. BMSCs and teno-iPSCs were infected with lentivirus expressing GFP (lenti-GFP) or equine Mohawk and GFP (lenti-MKX) for 5 days, and the whole cell lysates were blotted for MKX and *α*-tubulin.

Supplementary 7 Supplemental Figure 7 Effects of TGF-*β*3 or BMP12 on tenogenic gene expression in MKX-overexpressing iPSC1. Cells expressing GFP or equine Mohawk were treated with vehicle medium (GFP/BSA and MKX/BSA), TGF-*β*3 (20 ng/mL, GFP/TGF-*β*3 and MKX/TGF-*β*3), or BMP12 (20 ng/mL, GFP/BMP12 and MKX/BMP12) for 5 days. cDNA was synthesized from total RNA, and expression of tenogenic transcription factors (A), SOX9, RUNX2 (B) and tenocyte-related ECM genes (C) was determined by qPCR. Relative fold change for each group was calculated by comparison to the GFP-CTRL group. ^∗^Data were compared to the GFP/BSA group, and ^#^data were compared to the MKX/BSA group.

Supplementary 8 Supplemental Table 1 List of primer pairs used for qRT-PCR in this study.

## References

[1] Nourissat G., Berenbaum F., Duprez D. (2015). Tendon injury: from biology to tendon repair. *Nature Reviews Rheumatology*.

[2] Gaspar D., Spanoudes K., Holladay C., Pandit A., Zeugolis D. (2015). Progress in cell-based therapies for tendon repair. *Advanced Drug Delivery Reviews*.

[3] Bi Y., Ehirchiou D., Kilts T. M. (2007). Identification of tendon stem/progenitor cells and the role of the extracellular matrix in their niche. *Nature Medicine*.

[4] Tan Q., Lui P. P., Rui Y. F., Wong Y. M. (2012). Comparison of potentials of stem cells isolated from tendon and bone marrow for musculoskeletal tissue engineering. *Tissue Engineering. Part A*.

[5] Guo J., Chan K. M., Zhang J. F., Li G. (2016). Tendon-derived stem cells undergo spontaneous tenogenic differentiation. *Experimental Cell Research*.

[6] Webb S., Gabrelow C., Pierce J., Gibb E., Elliott J. (2016). Retinoic acid receptor signaling preserves tendon stem cell characteristics and prevents spontaneous differentiation in vitro. *Stem Cell Research & Therapy*.

[7] Harris M. T., Butler D. L., Boivin G. P., Florer J. B., Schantz E. J., Wenstrup R. J. (2004). Mesenchymal stem cells used for rabbit tendon repair can form ectopic bone and express alkaline phosphatase activity in constructs. *Journal of Orthopaedic Research*.

[8] Xu W., Wang Y., Liu E. (2013). Human iPSC-derived neural crest stem cells promote tendon repair in a rat patellar tendon window defect model. *Tissue Engineering. Part A*.

[9] Komura S., Satake T., Goto A. (2020). Induced pluripotent stem cell-derived tenocyte-like cells promote the regeneration of injured tendons in mice. *Scientific Reports*.

[10] Woods S., Bates N., Dunn S. L., Serracino-Inglott F., Hardingham T. E., Kimber S. J. (2019). Generation of human-induced pluripotent stem cells from anterior cruciate ligament. *Journal of Orthopaedic Research*.

[11] Gorecka J., Kostiuk V., Fereydooni A. (2019). The potential and limitations of induced pluripotent stem cells to achieve wound healing. *Stem Cell Research & Therapy*.

[12] Wolfman N. M., Hattersley G., Cox K. (1997). Ectopic induction of tendon and ligament in rats by growth and differentiation factors 5, 6, and 7, members of the TGF-beta gene family. *The Journal of Clinical Investigation*.

[13] Pryce B. A., Watson S. S., Murchison N. D., Staverosky J. A., Dunker N., Schweitzer R. (2009). Recruitment and maintenance of tendon progenitors by TGFbeta signaling are essential for tendon formation. *Development*.

[14] Barsby T., Guest D. (2013). Transforming growth factor beta3 promotes tendon differentiation of equine embryo-derived stem cells. *Tissue Engineering. Part A*.

[15] Tan G. K., Pryce B. A., Stabio A. (2020). Tgfbeta Signaling Is Critical for Maintenance of the Tendon Cell Fate. *eLife*.

[16] Chan K. M., Fu S. C., Wong Y. P., Hui W. C., Cheuk Y. C., Wong M. W. (2008). Expression of transforming growth factor beta isoforms and their roles in tendon healing. *Wound Repair and Regeneration*.

[17] Kapacee Z., Yeung C. Y., Lu Y., Crabtree D., Holmes D. F., Kadler K. E. (2010). Synthesis of embryonic tendon-like tissue by human marrow stromal/mesenchymal stem cells requires a three-dimensional environment and transforming growth factor *β*3. *Matrix Biology*.

[18] Bavin E. P., Smith O., Baird A. E., Smith L. C., Guest D. J. (2015). Equine induced pluripotent stem cells have a reduced tendon differentiation capacity compared to embryonic stem cells. *Frontiers in Veterinary Science*.

[19] Mikic B., Schalet B. J., Clark R. T., Gaschen V., Hunziker E. B. (2001). GDF-5 deficiency in mice alters the ultrastructure, mechanical properties and composition of the Achilles tendon. *Journal of Orthopaedic Research*.

[20] Mikic B., Rossmeier K., Bierwert L. (2009). Sexual dimorphism in the effect of GDF-6 deficiency on murine tendon. *Journal of Orthopaedic Research*.

[21] Mikic B., Bierwert L., Tsou D. (2006). Achilles tendon characterization in GDF-7 deficient mice. *Journal of Orthopaedic Research*.

[22] Violini S., Ramelli P., Pisani L. F., Gorni C., Mariani P. (2009). Horse bone marrow mesenchymal stem cells express embryo stem cell markers and show the ability for tenogenic differentiation by in vitro exposure to BMP-12. *BMC Cell Biology*.

[23] Gulati B. R., Kumar R., Mohanty N., Kumar P., Somasundaram R. K., Yadav P. S. (2013). Bone morphogenetic protein-12 induces tenogenic differentiation of mesenchymal stem cells derived from equine amniotic fluid. *Cells, Tissues, Organs*.

[24] Shen H., Gelberman R. H., Silva M. J., Sakiyama-Elbert S. E., Thomopoulos S. (2013). BMP12 induces tenogenic differentiation of adipose-derived stromal cells. *PLoS One*.

[25] Zarychta-Wisniewska W., Burdzinska A., Kulesza A. (2017). Bmp-12 activates tenogenic pathway in human adipose stem cells and affects their immunomodulatory and secretory properties. *BMC Cell Biology*.

[26] Chen X., Yin Z., Chen J. L. (2014). Scleraxis-overexpressed human embryonic stem cell-derived mesenchymal stem cells for tendon tissue engineering with knitted silk-collagen scaffold. *Tissue Engineering. Part A*.

[27] Otabe K., Nakahara H., Hasegawa A. (2015). Transcription factor Mohawk controls tenogenic differentiation of bone marrow mesenchymal stem cells in vitro and in vivo. *Journal of Orthopaedic Research*.

[28] Yang F., Zhang A., Richardson D. W. (2019). Regulation of the tenogenic gene expression in equine tenocyte-derived induced pluripotent stem cells by mechanical loading and Mohawk. *Stem Cell Research*.

[29] Gaut L., Robert N., Delalande A., Bonnin M. A., Pichon C., Duprez D. (2016). EGR1 regulates transcription downstream of mechanical signals during tendon formation and healing. *PLoS One*.

[30] Yin Z., Guo J., Wu T. Y. (2016). Stepwise differentiation of mesenchymal stem cells augments tendon-like tissue formation and defect repair in vivo. *Stem Cells Translational Medicine*.

[31] Vander Ark A., Cao J., Li X. (2018). TGF-*β* receptors: in and beyond TGF-*β* signaling. *Cellular Signalling*.

[32] Zhang Y. E. (2017). Non-Smad signaling pathways of the TGF-*β* family. *Cold Spring Harbor Perspectives in Biology*.

[33] Nakao A., Afrakhte M., Moren A. (1997). Identification of Smad7, a TGFbeta-inducible antagonist of TGF-beta signalling. *Nature*.

[34] Havis E., Bonnin M. A., Esteves de Lima J., Charvet B., Milet C., Duprez D. (2016). TGF*β* and FGF promote tendon progenitor fate and act downstream of muscle contraction to regulate tendon differentiation during chick limb development. *Development*.

[35] Murchison N. D., Price B. A., Conner D. A. (2007). Regulation of tendon differentiation by scleraxis distinguishes force-transmitting tendons from muscle-anchoring tendons. *Development*.

[36] Liu W., Watson S. S., Lan Y. (2010). The atypical homeodomain transcription factor Mohawk controls tendon morphogenesis. *Molecular and Cellular Biology*.

[37] Suzuki H., Ito Y., Shinohara M. (2016). Gene targeting of the transcription factor Mohawk in rats causes heterotopic ossification of Achilles tendon via failed tenogenesis. *Proceedings of the National Academy of Sciences of the United States of America*.

[38] Guerquin M. J., Charvet B., Nourissat G. (2013). Transcription factor EGR1 directs tendon differentiation and promotes tendon repair. *The Journal of Clinical Investigation*.

[39] Safi H. A., Nagalingam R. S., Czubryt M. P. (2018). Scleraxis: a force-responsive cell phenotype regulator. *Current Opinion in Physiology*.

[40] Brown J. P., Finley V. G., Kuo C. K. (2014). Embryonic mechanical and soluble cues regulate tendon progenitor cell gene expression as a function of developmental stage and anatomical origin. *Journal of Biomechanics*.

[41] Maeda T., Sakabe T., Sunaga A. (2011). Conversion of mechanical force into TGF-*β*-mediated biochemical signals. *Current Biology*.

[42] Liu H., Zhang C., Zhu S. (2015). Mohawk promotes the tenogenesis of mesenchymal stem cells through activation of the TGF*β* signaling pathway. *Stem Cells*.

[43] Koda N., Sato T., Shinohara M. (2017). The transcription factor Mohawk homeobox regulates homeostasis of the periodontal ligament. *Development*.

[44] Ito Y., Toriuchi N., Yoshitaka T. (2010). The Mohawk homeobox gene is a critical regulator of tendon differentiation. *Proceedings of the National Academy of Sciences*.

[45] Kayama T., Mori M., Ito Y. (2016). Gtf2ird1-dependent Mohawk expression regulates mechanosensing properties of the tendon. *Molecular and Cellular Biology*.

[46] Lee J. Y., Zhou Z., Taub P. J. (2011). BMP-12 treatment of adult mesenchymal stem cells in vitro augments tendon-like tissue formation and defect repair in vivo. *PLoS One*.

[47] Wang Q. W., Chen Z. L., Piao Y. R. (2005). Mesenchymal stem cells differentiate into tenocytes by bone morphogenetic protein (BMP) 12 gene transfer. *Journal of Bioscience and Bioengineering*.

[48] Lejard V., Blais F., Guerquin M. J. (2011). EGR1 and EGR2 involvement in vertebrate tendon differentiation. *The Journal of Biological Chemistry*.

[49] Liu Q., Zhu Y., Qi J. (2019). Isolation and characterization of turkey bone marrow-derived mesenchymal stem cells. *Journal of Orthopaedic Research*.

[50] Fahy N., Alini M., Stoddart M. J. (2018). Mechanical stimulation of mesenchymal stem cells: implications for cartilage tissue engineering. *Journal of Orthopaedic Research*.

[51] Screen H. R., Berk D. E., Kadler K. E., Ramirez F., Young M. F. (2015). Tendon functional extracellular matrix. *Journal of Orthopaedic Research*.

[52] Ramirez F., Tanaka S., Bou-Gharios G. (2006). Transcriptional regulation of the human alpha2(I) collagen gene (COL1A2), an informative model system to study fibrotic diseases. *Matrix Biology*.

[53] Thorpe C. T., Birch H. L., Clegg P. D., Screen H. R. (2013). The role of the non-collagenous matrix in tendon function. *International Journal of Experimental Pathology*.

[54] Zhang W., Ge Y., Cheng Q., Zhang Q., Fang L., Zheng J. (2018). Decorin is a pivotal effector in the extracellular matrix and tumour microenvironment. *Oncotarget*.

[55] Youngstrom D. W., LaDow J. E., Barrett J. G. (2016). Tenogenesis of bone marrow-, adipose-, and tendon-derived stem cells in a dynamic bioreactor. *Connective Tissue Research*.

[56] Lohberger B., Kaltenegger H., Stuendl N., Rinner B., Leithner A., Sadoghi P. (2016). Impact of cyclic mechanical stimulation on the expression of extracellular matrix proteins in human primary rotator cuff fibroblasts. *Knee Surgery, Sports Traumatology, Arthroscopy*.

[57] Nichols A. E. C., Werre S. R., Dahlgren L. A. (2018). Transient scleraxis overexpression combined with cyclic strain enhances ligament cell differentiation. *Tissue Engineering. Part A*.

[58] Xu S. Y., Liu S. Y., Xu L. (2018). Response of decorin to different intensity treadmill running. *Molecular Medicine Reports*.

[59] Svensson L., Aszodi A., Reinholt F. P., Fassler R., Heinegard D., Oldberg A. (1999). Fibromodulin-null mice have abnormal collagen fibrils, tissue organization, and altered lumican deposition in tendon. *The Journal of Biological Chemistry*.

[60] Xu Y., Wang Q., Li Y. D. (2015). Cyclic tensile strain induces tenogenic differentiation of tendon-derived stem cells in bioreactor culture. *Biomed Research International*.

[61] Grant T. M., Thompson M. S., Urban J., Yu J. (2013). Elastic fibres are broadly distributed in tendon and highly localized around tenocytes. *Journal of Anatomy*.

[62] Wu Y. T., Su W. R., Wu P. T., Shen P. C., Jou I. M. (2017). Degradation of elastic fiber and elevated elastase expression in long head of biceps tendinopathy. *Journal of Orthopaedic Research*.

[63] Thakkar D., Grant T. M., Hakimi O., Carr A. J. (2014). Distribution and expression of type VI collagen and elastic fibers in human rotator cuff tendon tears. *Connective Tissue Research*.

[64] Min J., Li B., Liu C. (2017). Extracellular matrix metabolism disorder induced by mechanical strain on human parametrial ligament fibroblasts. *Molecular Medicine Reports*.

[65] Mackie E. J., Ramsey S. (1996). Expression of tenascin in joint-associated tissues during development and postnatal growth. *Journal of Anatomy*.

[66] Taylor S. E., Vaughan-Thomas A., Clements D. N. (2009). Gene expression markers of tendon fibroblasts in normal and diseased tissue compared to monolayer and three dimensional culture systems. *BMC Musculoskeletal Disorders*.

[67] Jarvinen T. A., Jozsa L., Kannus P. (2003). Mechanical loading regulates the expression of tenascin-C in the myotendinous junction and tendon but does not induce de novo synthesis in the skeletal muscle. *Journal of Cell Science*.

[68] Qiu Y., Lei J., Koob T. J., Temenoff J. S. (2016). Cyclic tension promotes fibroblastic differentiation of human MSCs cultured on collagen-fibre scaffolds. *Journal of Tissue Engineering and Regenerative Medicine*.

[69] Roth S. P., Brehm W., Gross C., Scheibe P., Schubert S., Burk J. (2019). Transforming growth factor beta 3-loaded decellularized equine tendon matrix for orthopedic tissue engineering. *International Journal of Molecular Sciences*.

[70] Shukunami C., Takimoto A., Nishizaki Y. (2018). Scleraxis is a transcriptional activator that regulates the expression of tenomodulin, a marker of mature tenocytes and ligamentocytes. *Scientific Reports*.

[71] Molloy T., Wang Y., Murrell G. (2003). The roles of growth factors in tendon and ligament healing. *Sports Medicine*.

[72] Lim J., Munivez E., Jiang M. M. (2017). mTORC1 signaling is a critical regulator of postnatal tendon development. *Scientific Reports*.

[73] Cong X. X., Rao X. S., Lin J. X. (2018). Activation of AKT-mTOR signaling directs tenogenesis of mesenchymal stem cells. *Stem Cells*.

[74] Kishimoto Y., Ohkawara B., Sakai T. (2017). Wnt/*β*-catenin signaling suppresses expressions of Scx, Mkx, and Tnmd in tendon-derived cells. *PLoS One*.

[75] Bitzer M., von Gersdorff G., Liang D. (2000). A mechanism of suppression of TGF-beta/SMAD signaling by NF-kappa B/RelA. *Genes & Development*.

[76] Weng H., Mertens P. R., Gressner A. M., Dooley S. (2007). IFN-gamma abrogates profibrogenic TGF-beta signaling in liver by targeting expression of inhibitory and receptor Smads. *Journal of Hepatology*.

[77] Kim S., Han J., Lee S. K. (2012). Smad7 acts as a negative regulator of the epidermal growth factor (EGF) signaling pathway in breast cancer cells. *Cancer Letters*.

[78] O'Hagan R. C., Tozer R. G., Symons M., McCormick F., Hassell J. A. (1996). The activity of the Ets transcription factor PEA3 is regulated by two distinct MAPK cascades. *Oncogene*.

[79] Paxton J. Z., Hagerty P., Andrick J. J., Baar K. (2012). Optimizing an intermittent stretch paradigm using ERK1/2 phosphorylation results in increased collagen synthesis in engineered ligaments. *Tissue Engineering. Part A*.

[80] Nishimura K., Blume P., Ohgi S., Sumpio B. E. (2009). The effect of different frequencies of stretch on human dermal keratinocyte proliferation and survival. *The Journal of Surgical Research*.

[81] Busch F., Mobasheri A., Shayan P., Stahlmann R., Shakibaei M. (2012). Sirt-1 is required for the inhibition of apoptosis and inflammatory responses in human tenocytes. *The Journal of Biological Chemistry*.

[82] Lee S. I., Park K. H., Kim S. J., Kang Y. G., Lee Y. M., Kim E. C. (2012). Mechanical stress-activated immune response genes via Sirtuin 1 expression in human periodontal ligament cells. *Clinical and Experimental Immunology*.

[83] Zhang C., Zhang E., Yang L. (2018). Histone deacetylase inhibitor treated cell sheet from mouse tendon stem/progenitor cells promotes tendon repair. *Biomaterials*.

[84] Wang D., Jiang X., Lu A., Tu M., Huang W., Huang P. (2018). BMP14 induces tenogenic differentiation of bone marrow mesenchymal stem cells <i>in vitro</i>. *Experimental and Therapeutic Medicine*.

[85] Zerr P., Palumbo-Zerr K., Huang J. (2015). Sirt1 regulates canonical TGF-beta signalling to control fibroblast activation and tissue fibrosis. *Annals of the Rheumatic Diseases*.

