# Supplementary figures and images for: Comparative Analysis of Tenogenic Gene Expression in Tenocyte-Derived Induced Pluripotent Stem Cells and Bone Marrow-Derived Mesenchymal Stem Cells in Response to Biochemical and Biomechanical Stimuli
Source: Stem Cells Int. 2021 Jan 13;2021:8835576. doi: 10.1155/2021/8835576 (PMC7825360; doi:10.1155/2021/8835576)

Supplemental Figure 1

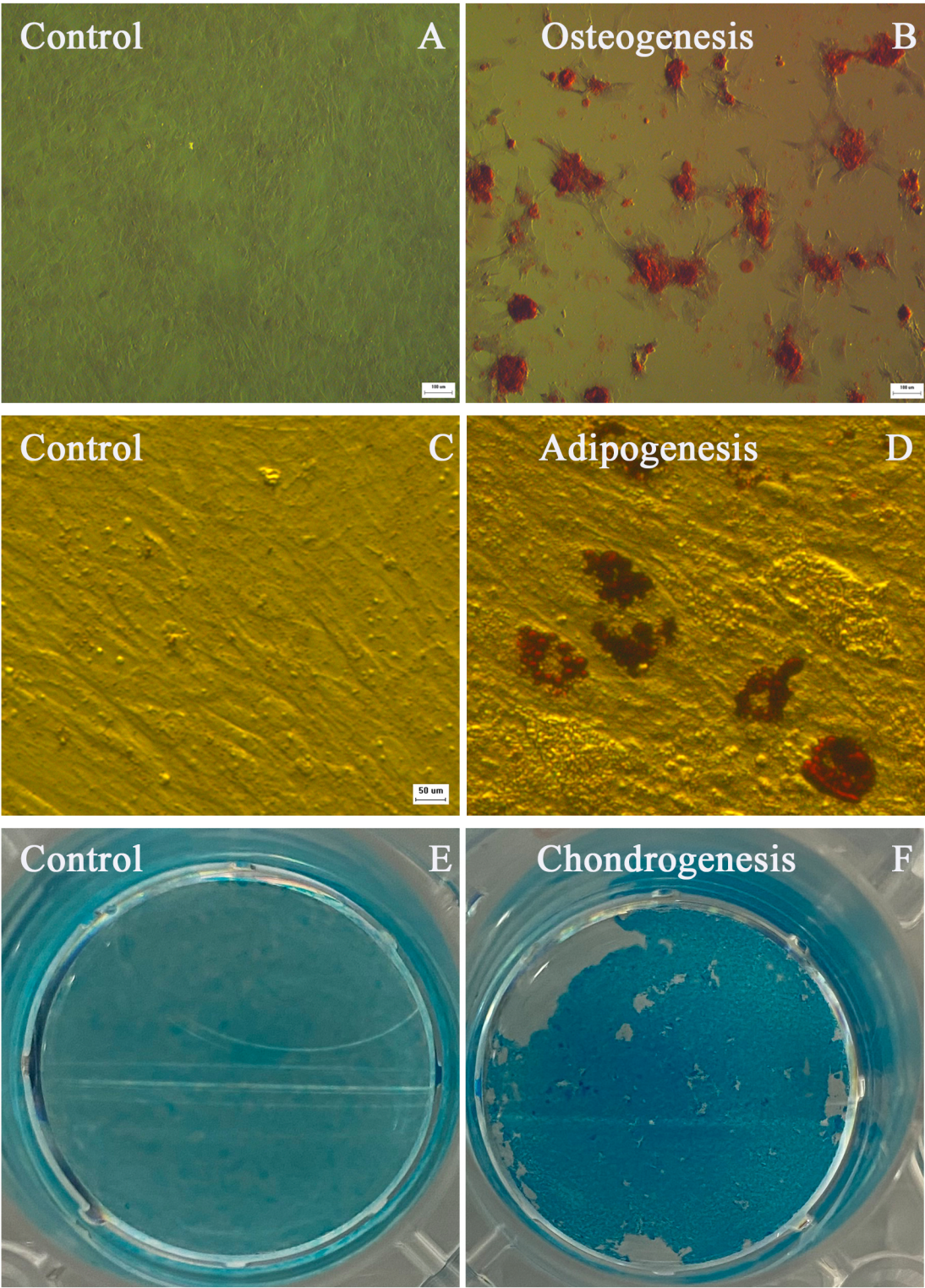

Supplement: Supplementary 1 — Supplemental Figure 1 Multilineage differentiation capacity of BMSCs. A, B In vitro osteogenic differentiation of BMSCs. The calcium deposition was revealed by Alizarin Red S staining. C, D In vitro adipogenic differentiation of BMSCs. The fat droplets were displayed by oil-red staining. E, F In vitro chondrogenic differentiation of teno-iPSCs. The production of proteoglycan proteins was shown by Alcian blue staining. [file 8835576.f1.pdf]

Supplemental Figure 3

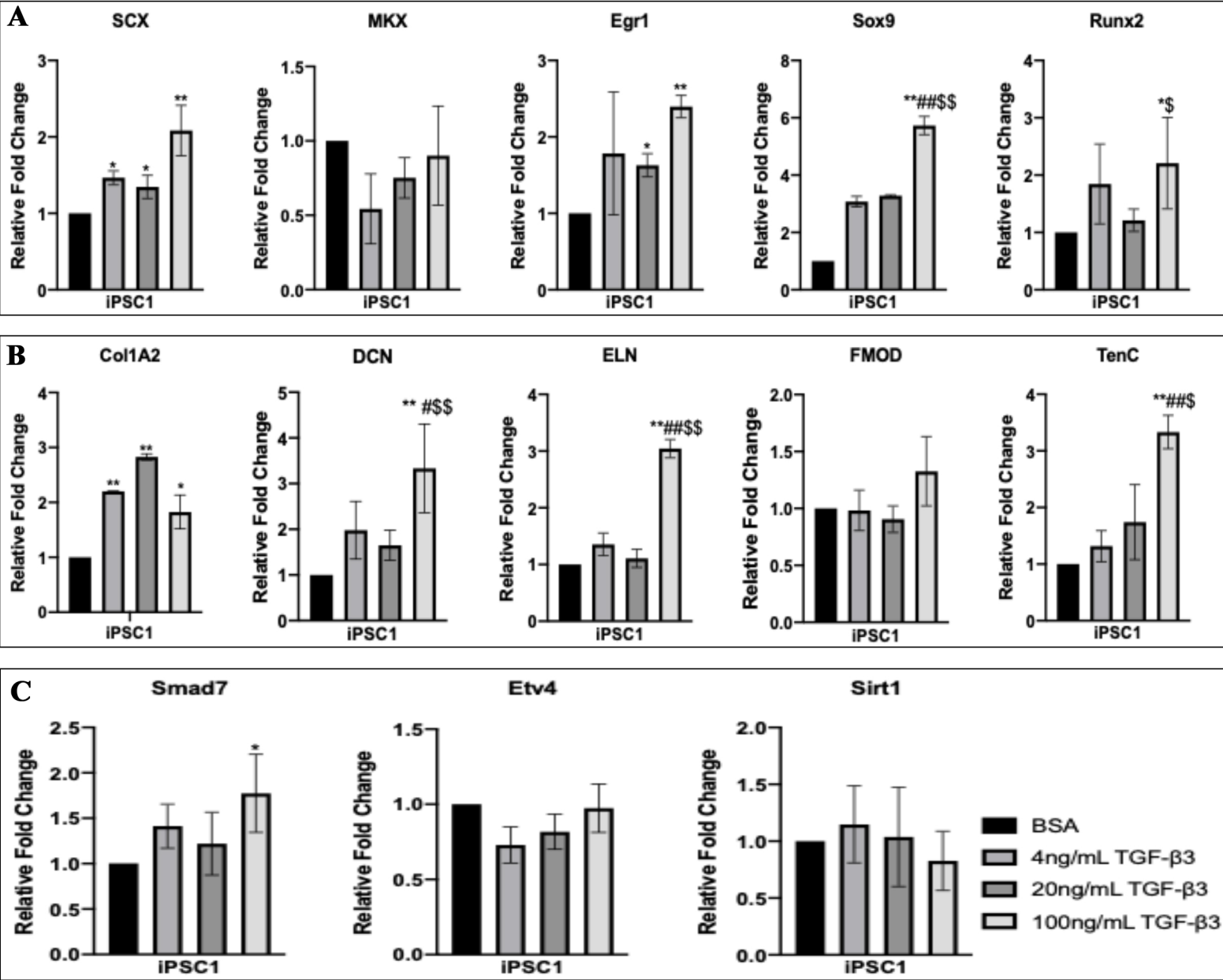

Supplement: Supplementary 3 — Supplemental Figure 3 Dose effects of TGF-β3 on tenogenic gene expression in iPSC1. iPSC1 were treated with vehicle medium (0) or various concentrations of TGF-β3 (4, 20, and 100 ng/mL) for 5 days, and cDNA was synthesized from total RNA. Expression of tenogenic transcription factors (A), chondrogenic transcription factor Sox9, osteogenic transcription factor RUNX2 (B), and tenocyte-related ECM genes (C) was determined by qPCR. Relative fold change for each group was calculated by comparison to vehicle medium group, and data were summarized from 3 passages. ∗Data were compared to BSA control; #data were compared to the 4 ng/mL group; $data were compared to 20 ng/mL group. [file 8835576.f3.pdf]

**Supplemental Figure 4**

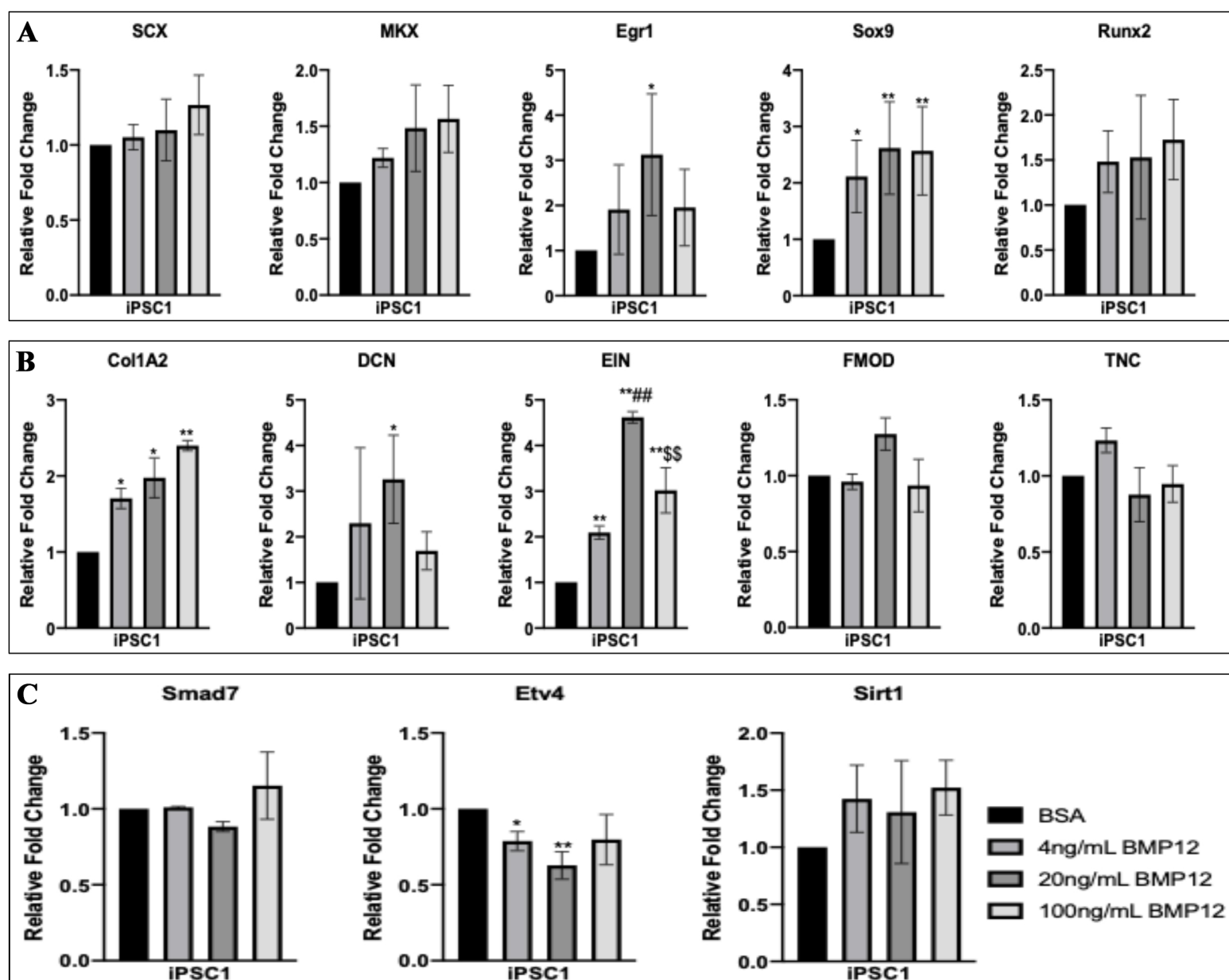

Supplement: Supplementary 4 — Supplemental Figure 4 Dose effects of BMP12 on tenogenic gene expression in iPSC1. Cells were treated with vehicle medium (0) or various concentrations of BMP12 (4, 20, and 100 ng/mL) for 5 days, and cDNA was synthesized from total RNA. Expression of tenogenic transcription factors (A), SOX9, RUNX2 (B), and tenocyte-related ECM genes (C) was determined by qPCR. Relative fold change for each group was calculated by comparison to the vehicle medium group, and data were summarized from 3 passages. ∗Data were compared to BSA control; #data were compared to the 4 ng/mL group; $data were compared to the 20 ng/mL group. [file 8835576.f4.pdf]

Supplemental Figure 5

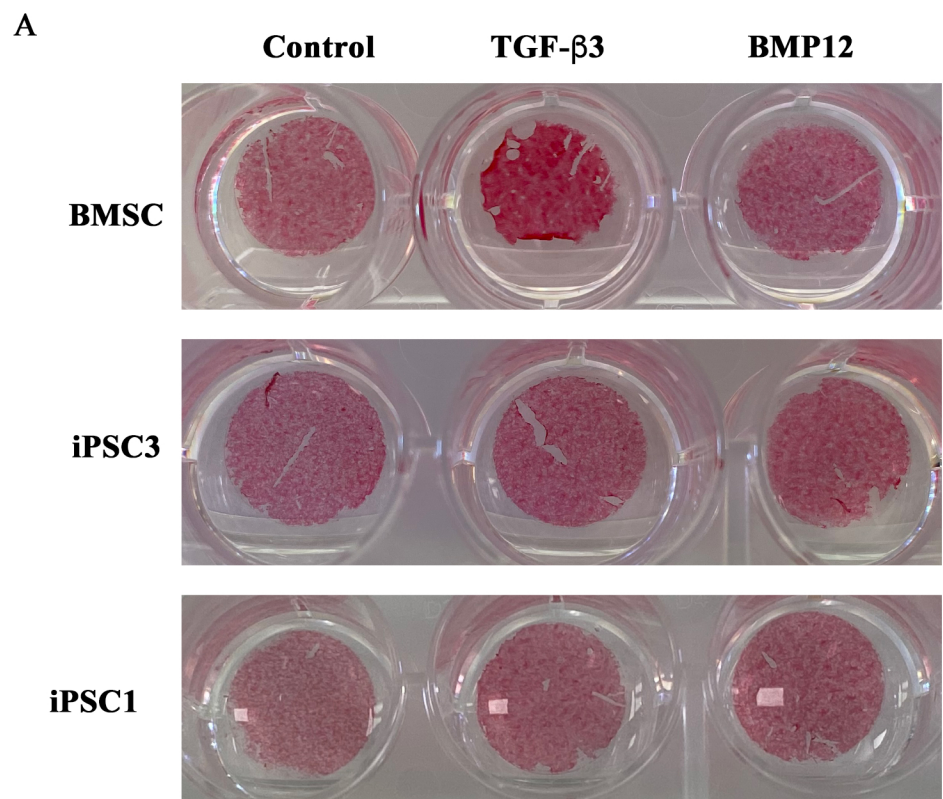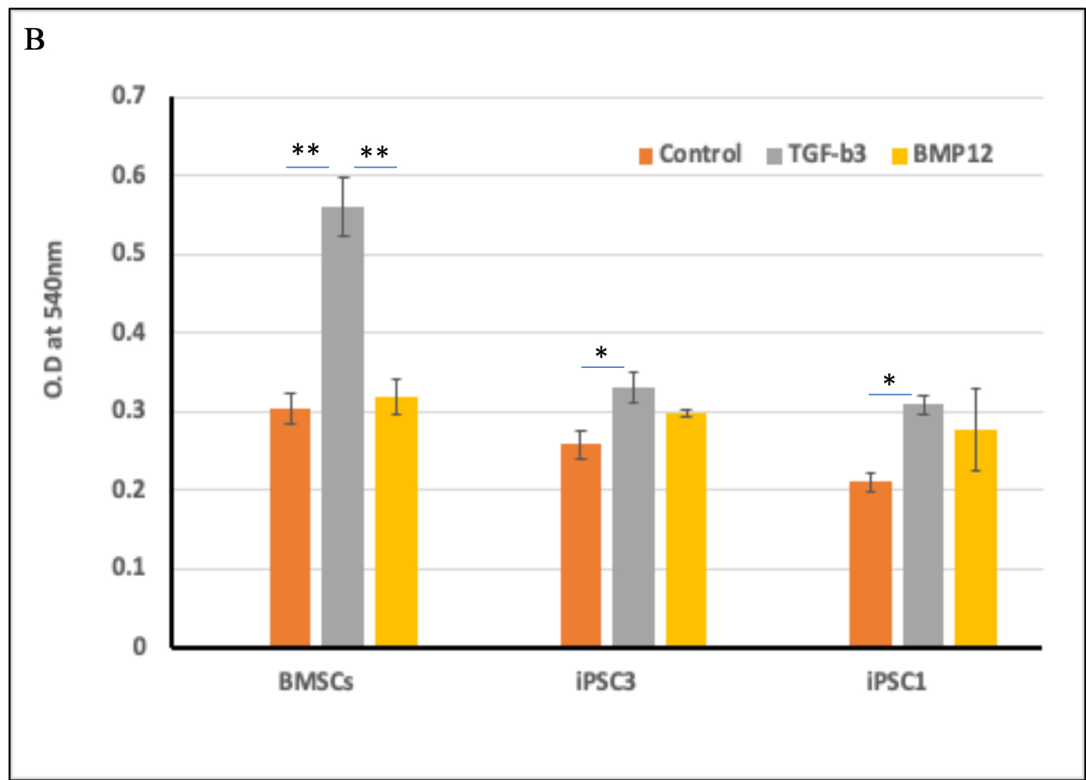

Supplement: Supplementary 5 — Supplemental Figure 5 Sirius red staining in TGF-β3- and BMP12-treated teno-iPSCs and BMSCs. A Cells were treated with TGF-β3 and BMP12 for 5 days, then fixed and stained with Sirius red. B Quantitation of Sirius red staining. ∗p < 0.05; ∗∗p < 0.01. [file 8835576.f5.pdf]

**Supplemental Figure 6**

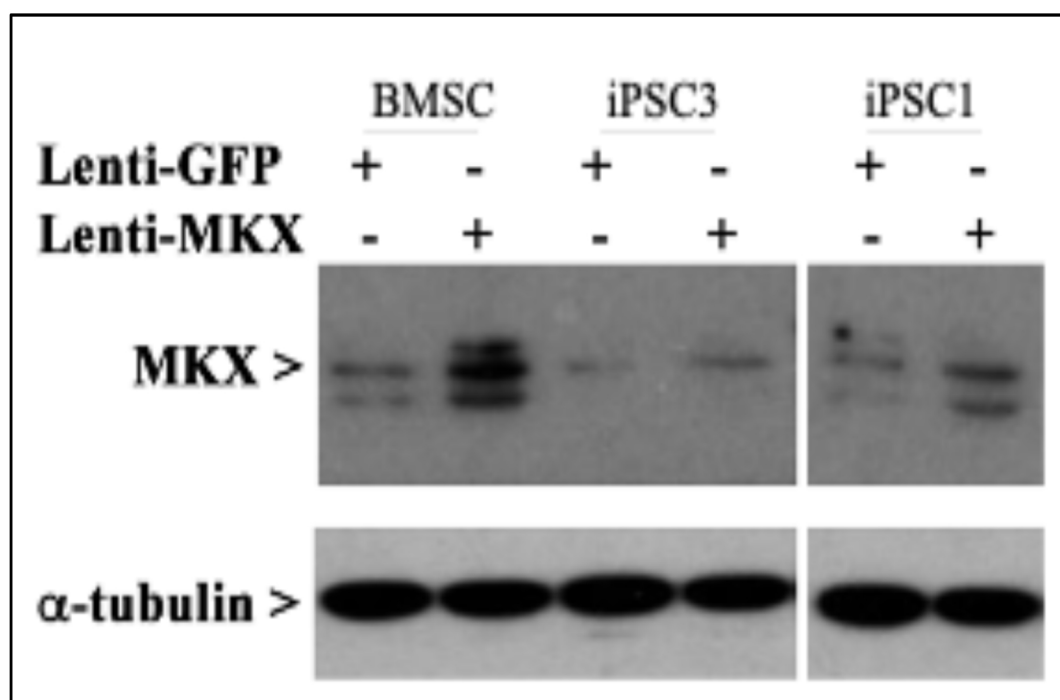

Supplement: Supplementary 6 — Supplemental Figure 6 Overexpression of MKX in BMSCs and teno-iPSCs. BMSCs and teno-iPSCs were infected with lentivirus expressing GFP (lenti-GFP) or equine Mohawk and GFP (lenti-MKX) for 5 days, and the whole cell lysates were blotted for MKX and α-tubulin. [file 8835576.f6.pdf]

Supplemental Figure 7

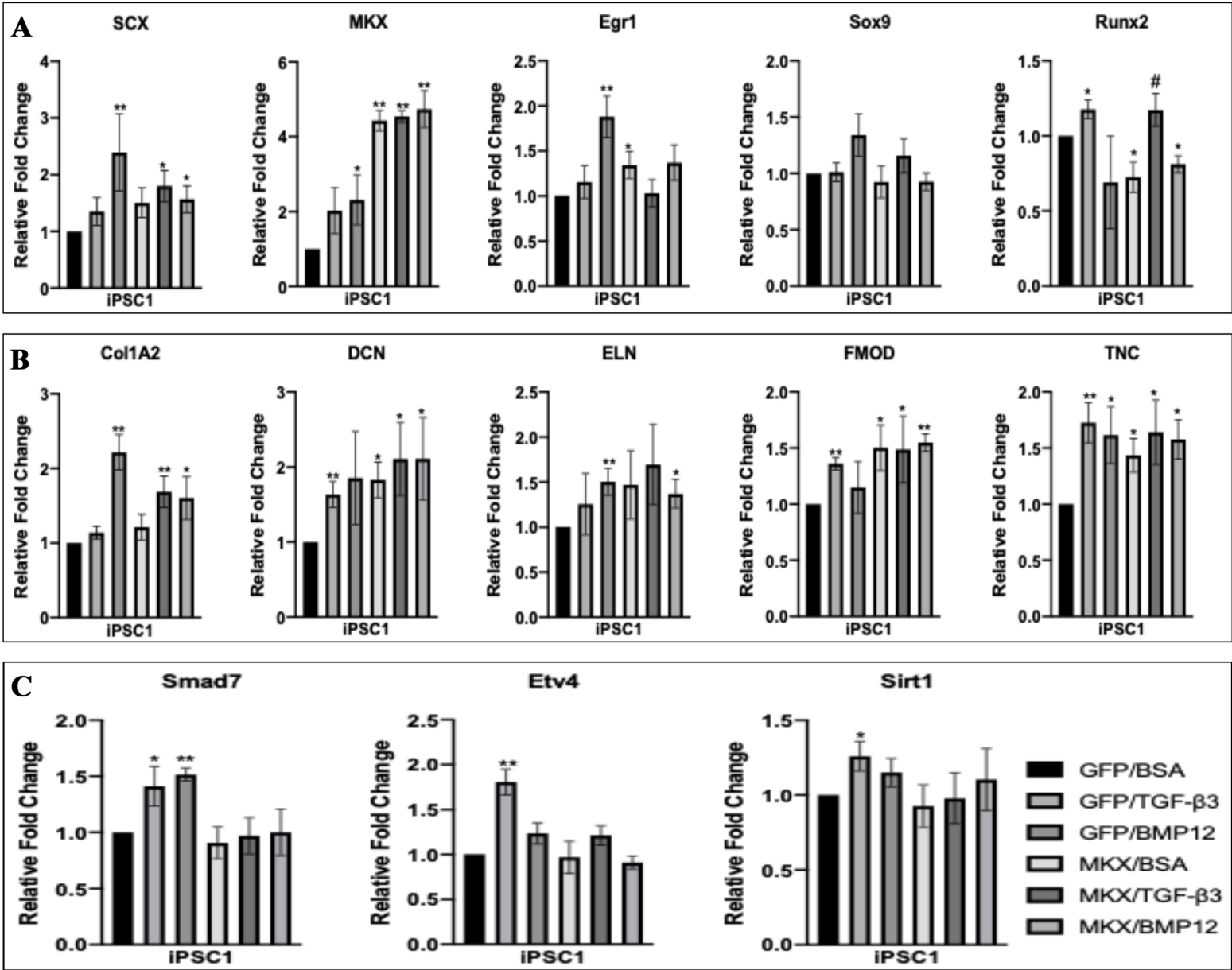

Supplement: Supplementary 7 — Supplemental Figure 7 Effects of TGF-β3 or BMP12 on tenogenic gene expression in MKX-overexpressing iPSC1. Cells expressing GFP or equine Mohawk were treated with vehicle medium (GFP/BSA and MKX/BSA), TGF-β3 (20 ng/mL, GFP/TGF-β3 and MKX/TGF-β3), or BMP12 (20 ng/mL, GFP/BMP12 and MKX/BMP12) for 5 days. cDNA was synthesized from total RNA, and expression of tenogenic transcription factors (A), SOX9, RUNX2 (B) and tenocyte-related ECM genes (C) was determined by qPCR. Relative fold change for each group was calculated by comparison to the GFP-CTRL group. ∗Data were compared to the GFP/BSA group, and #data were compared to the MKX/BSA group. [file 8835576.f7.pdf]
