# Supplementary material for: Comparative Analysis of Tenogenic Gene Expression in Tenocyte-Derived Induced Pluripotent Stem Cells and Bone Marrow-Derived Mesenchymal Stem Cells in Response to Biochemical and Biomechanical Stimuli
Source: Stem Cells Int. 2021 Jan 13;2021:8835576. doi: 10.1155/2021/8835576 (PMC7825360; doi:10.1155/2021/8835576)

Supplemental Figure 2

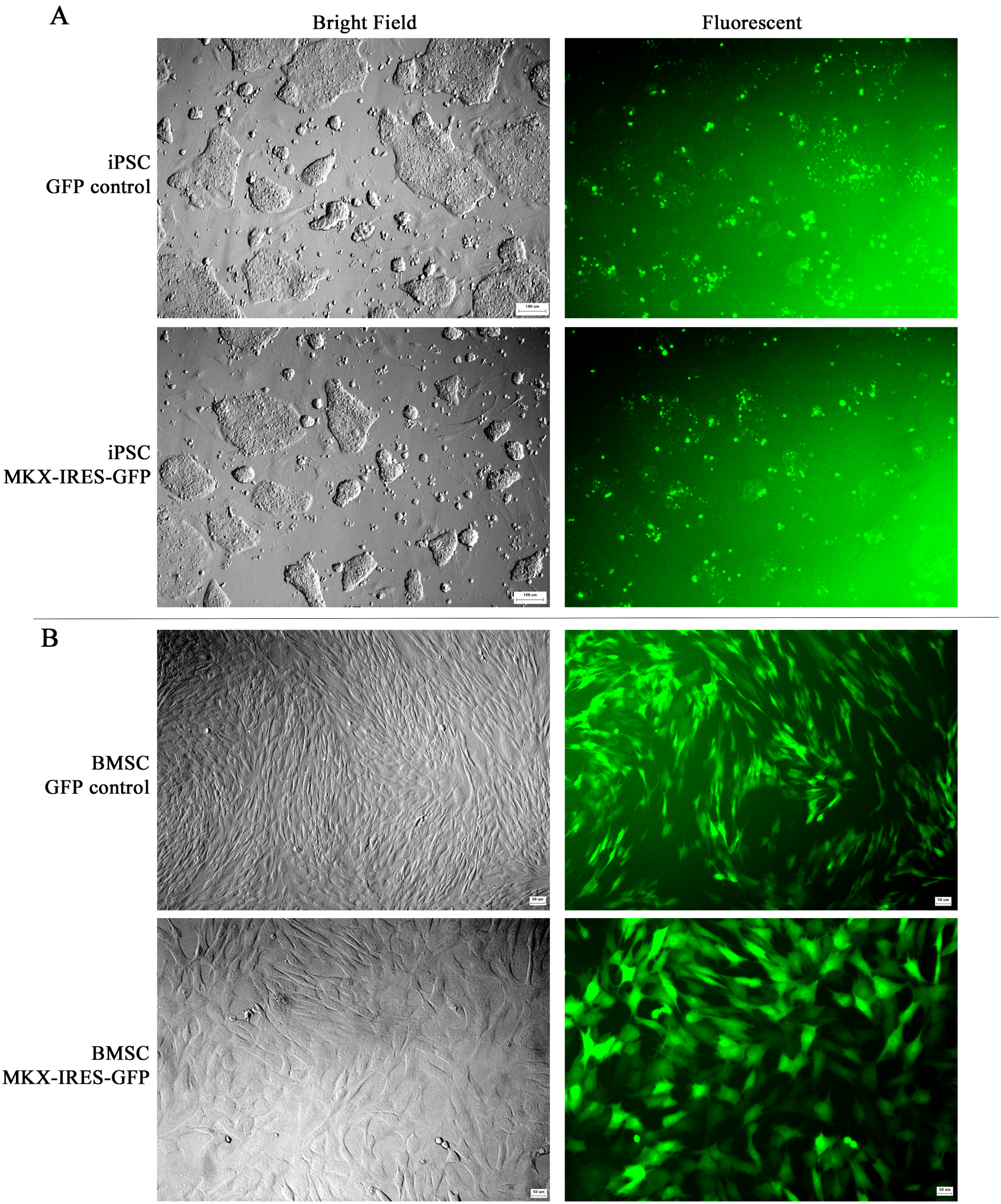

Supplement: Supplementary 2 — Supplemental Figure 2 Expression of GFP in lentiviral-infected teno-iPSCs and BMSCs. Teno-iPSCs (A) and BMSCs (B) were infected with lentivirus expressing GFP alone or MKX and GFP for 5 days. GFP signal was imaged under fluorescent microscope. [file 8835576.f2.pdf]
