# Supplementary material for: Comparative Analysis of Tenogenic Gene Expression in Tenocyte-Derived Induced Pluripotent Stem Cells and Bone Marrow-Derived Mesenchymal Stem Cells in Response to Biochemical and Biomechanical Stimuli
Source: Stem Cells Int. 2021 Jan 13;2021:8835576. doi: 10.1155/2021/8835576 (PMC7825360; doi:10.1155/2021/8835576)
Supplement: Supplementary 8 — Supplemental Table 1 List of primer pairs used for qRT-PCR in this study. [file 8835576.f8.docx]

# Supplemental Table 1: List of primer pairs used for qRT-PCR in this study

| Gene Name | | Sequence (5’ 🡪 3’) | Product size  (bp) |
| --- | --- | --- | --- |
| Col1A2 | For: | CGGGAGGTTTCGGCTAAGTT | 218 |
|  | Rev: | TTCCTGCAGTTGCCTCTTGT |  |
| Decorin  (*DCN)* | For: | TTATCAAAGTGCCTGGTG | 204 |
|  | Rev: | CATAGACACATCGGAAGG |  |
| Egr1 | For: | CCTACGAGCACCTGACCTCAG | 241 |
|  | Rev: | GATGGTGCTGAAGATGAAGTGG |  |
| Elastin (*ELN*) | For: | CTATGGTGTCGGTGTCGGAG | 247 |
|  | Rev: | GGGGGCTAACCCAAACTGAG |  |
| Etv4 | For: | GCAAGTGCCCTACACCTTCT | 238 |
|  | Rev: | GGGGCTGTGGAAAGCTAAGT |  |
| FMOD | For: | GCTTCTGCTGAGGGACAC | 90 |
|  | Rev: | GATTTCTGGGGTTGGGAC |  |
| Mohawk  (*MKX*) | For: | TAATCCCGTTCACCATCC | 195 |
|  | Rev: | CTTTGCCTTGTCTTTCCC |  |
| PSMB2 | For: | CGCCAGCATGGAGTACCTTA | 160 |
|  | Rev: | GCCAGCCTCTCCAACACATA |  |
| RUNX2 | For: | GAACCCAGAAGGCACAGACA | 250 |
|  | Rev: | GGCTCAGGTAGGAGGGGTAA |  |
| Scleraxis (*SCX*) | For: | CCCCCACGGACCTGACTC | 167 |
|  | Rev: | GGTAGGAAGCCAGCACGG |  |
| Sirt1 | For: | GATAGAGCCTCACATGCAAGC | 298 |
|  | Rev: | AAACCCCAGCTCCAGTTAGAA |  |
| Smad7 | For: | CAGGCATTCCTCGGAAGTCA | 174 |
|  | Rev: | GGACAGTCTGCAGTTGGTTTG |  |
| Sox9 | For: | CTGGAGACTGCTGAACGAGA | 171 |
|  | Rev: | GAGATGTGTGTCTGCTCCGT |  |
| Tenascin C  (*TNC*) | For: | GAACACGGTGGAGTATGC | 105 |
|  | Rev: | TTGGTAGTGATGGCTGAG |  |
| TNMD#1 | For: | GGCGGGTTATCTGTCGTG | 169 |
|  | Rev: | TACCAGGAGCCAAATGCC |  |
| TNMD#5 | For: | TCAGTGATTTGGGTCCCAGC | 229 |
|  | Rev: | CCTTCACTTGAGGGACCACC |  |
| TNMD#7 | For: | TGAACAAAACGAGCAGTGGG | 242 |
|  | Rev: | GCCCTCCTTGGTAGCAGTAT |  |
